# Magnitudes of Various Forms of Undernutrition Among Children from the Composite Index of Anthropometric Failure in Sub-Saharan Africa: A Systematic Review and Meta-Analysis

**DOI:** 10.3390/nu17111818

**Published:** 2025-05-27

**Authors:** Misganaw Gebrie Worku, Itismita Mohanty, Zelalem Mengesha, Theo Niyonsenga

**Affiliations:** 1 Health Research Institute, Faculty of Health, University of Canberra, Canberra, ACT 2617, Australia; 2 Department of Human Anatomy, School of Medicine, College of Medicine and Health Sciences, University of Gondar, Gondar P.O. Box 196, Ethiopia

**Keywords:** anthropometry, CIAF, coexisting, malnutrition, undernutrition

## Abstract

**Background:** Nearly half of under-five deaths are linked to undernutrition. Most evidence on undernutrition relies on conventional anthropometric measures. Conventional anthropometric measures fail to capture its overlapping forms and are limited in providing the true burden and distinct disaggregated patterns of undernutrition. Using the Composite Index of Anthropometric Failure (CIAF), this study aims to provide updated regional and country-level pooled prevalence estimates of the overall burden and various single and coexisting patterns of undernutrition among children in Sub-Saharan Africa (SSA). **Methods**: We systematically searched Medline, CINAHL, Web of Science, Scopus, and Google Scholar for studies published between January 2006 and October 2023. Studies reporting the prevalence of aggregated CIAF or any of the disaggregated forms of CIAF (stunting only, wasting only, underweight only, stunting-underweight, wasting-underweight, and stunting-wasting-underweight) based on the 2006 World Health Organisation (WHO) growth standard were included. Data extraction was performed by two reviewers, and discrepancies were resolved by consensus. Pooled prevalences of various categories of undernutrition were estimated using a random effect model meta-analysis. Subgroup analysis and meta-regression were performed to identify possible sources of heterogeneity among the included studies. Publication bias was checked using the Asymmetry funnel plot and Egger’s test. The protocol was registered on PROSPERO (CRD42023458796). **Result**: This systematic review and meta-analysis identified 3898 published studies from the database search, of which 26 were included. In SSA, the overall pooled prevalence of undernutrition among children was 37.45% (95% Confidence Interval (CI): 31.97, 42.92). Of these, 10% (95% CI: 8.02, 11.98) of children experienced at least one coexisting form, and 25.5% (95% CI: 16.78, 33.72) experienced at least one single form of undernutrition. Stunting only [22.32% (95% CI: 18.26, 26.39)] was the most prevalent disaggregated pattern of undernutrition, followed by the coexistence of stunting with underweight [10.15% (95% CI: 8.17, 12.13)]. **Conclusions**: Over one in three children in SSA experienced at least one form of undernutrition. Nearly one-third of these undernourished children were affected by multiple forms of undernutrition. The high prevalence of coexisting undernutrition indicates the need to develop multi-indicator nutrition strategies that could simultaneously address the various dimensions of undernutrition in children.

## 1. Introduction

Undernutrition contributes to nearly half of deaths among children under five years of age [1]. Ensuring proper child nutrition is essential for fostering holistic growth and development. Conversely, undernutrition hinders both child growth and economic development. Furthermore, various forms and patterns of undernutrition significantly affect the burden of communicable diseases and the attainment of early child development milestones [2,3]. Thus, undernutrition plays a significant role in shaping multiple aspects of child health.

The available evidence on undernutrition of children primarily relies on conventional measurements such as stunting (height for age < −2 standard deviation (SD) from the median of the world health organisation (WHO) Child Growth Standards), wasting (weight for height < −2 SD), and underweight (weight for age < −2 SD) [4,5,6,7]. Undernutrition measurement from the conventional methods addresses only limited dimensions of undernutrition. Additionally, these conventional methods fail to provide a comprehensive view of the actual burden of undernutrition among children. A recent study has highlighted that the challenges of undernutrition extend beyond these conventional indicators, as many children experience combinations of different forms of undernutrition simultaneously [8]. Furthermore, children with double and triple anthropometric deficits face markedly higher risks of morbidity and mortality [9]. While children suffering from single forms of undernutrition have 2 to 3 times higher risk of morbidity and mortality compared to well-nourished children, these risks can rise up to 10 to 15 folds among children affected by coexisting forms of undernutrition [9]. This highlights that children with single and coexisting forms of undernutrition experience distinct health implications.

Various methods of undernutrition classifications are available to estimate the magnitude and severity of under-five undernutrition. The most widely used classification is based on weight-for-age measurement, also known as the Indian Academy of Pediatrics (IAP) Gomez classification [10,11]. The McLaren Waterlow classification, which utilises height for age and weight for height, is the second most commonly used undernutrition classification [10]. Those conventional classification systems fail to identify coexisting forms of undernutrition because they typically assess each form (wasting, stunting, and underweight) independently. These systems disregard the overlapping or combined effects of multiple deficiencies. Moreover, specific identification of each facet of child undernutrition is crucial for priority setting in policy and clinical practice. Thus, relying solely on conventional undernutrition measurements overlooks critical aspects of undernutrition, leading to gaps in policy practice.

The Composite Index of Anthropometric Failure (CIAF), originally proposed by Peter Svedberg and afterward refined by Nandy et al., offers a comprehensive method for assessing the burden and severity of child undernutrition [12,13]. CIAF in the aggregated form helps to obtain estimates of the overall prevalence of undernutrition, while in its disaggregated forms, it helps to identify and obtain estimates for the various single, double, and triple forms of undernutrition. Utilising CIAF as a measurement tool for child undernutrition could resolve the issue of non-mutually exclusivity among conventional indices by introducing new categories of undernutrition [3,14]. CIAF is currently the only approach capable of simultaneously accounting for all forms of child undernutrition and can specifically identify children affected by single and coexisting forms of undernutrition.

Reliance of primary organisations, including the WHO, on stunting and wasting alone leads to underestimation of total nutritional burden and conceals overlapping forms. This potentially hinders the development of effective nutritional policies, programmes, and evaluation of progress made so far [15,16].

Despite the CIAF benefit in comprehensively assessing child undernutrition, there is a lack of systemic analysis of available evidence on the various forms of undernutrition from the CIAF. Therefore, we conducted a systematic review and meta-analysis to systematically synthesise undernutrition prevalence evidence from the CIAF and explore the patterns of undernutrition among children in SSA.

## 2. Methods

### 2.1. Searching Strategies and Screening

We conducted a systematic review and meta-analysis following the Preferred Reporting Items for Systematic Review and Meta-Analysis (PRISMA) guidelines. The protocol was registered on the International Prospective Register of Systematic Reviews (PROSPERO) with a registration number CRD42023458796. We systematically searched Medline (EBSCOhost), CINAHL (EBSCOhost), Scopus, Web of Science, and Google Scholar to identify the relevant literature. A combination of keywords and/or MeSH terms were used for searching, and Boolean operators (OR, AND, and NOT) were used to combine search terms. Wildcards and truncations were inserted according to the database used. Additionally, reference lists of included studies were searched for the additional literature. Below is the full search term of the Web of Science database, with the full details of other database searches provided in Supplementary File S1.

The search term (prevalence OR magnitude OR burden OR estimate* OR “overall burden” OR “pooled prevalence” OR “pooled magnitude” (topic) AND malnutrition* OR malnourish* OR undernutrition* OR undernourish* OR “nutritional outcome*” OR “nutritional deficien*” OR “nutritional disorder” OR “insufficient nutrition” OR “nutritional status” OR “nutritional problem*” OR starvation* OR hunger OR stunt* OR “height for age” OR “length for age” OR wast* OR thinness OR “weight for height” OR underweight* OR “weight for age” OR “stunting index” OR “wasting index” OR “underweight index” OR “undernutrition ind*” OR (composite OR conventional) NEAR/1 (failure OR ind*) OR “anthropometric deficien*” OR (standalone OR single OR double OR triple OR multiple OR coexisting) NEAR/3 (failure OR undernutrition) OR “multiple undernutrition” OR “coexisting undernutrition” OR “concurr* undernutrition” OR “overlapped undernutrition” OR “anthropometric assessment*” OR “anthropometric evaluation” (topic) AND child* OR “young child” OR infan* OR neona* OR toddler* OR p$ediatric* OR bab* OR under NEAR/3 (five OR two) OR preschool OR kid* OR “0 to 60 month*” OR “0 to 59 month*” OR “6 to 24 month*” OR “6 to 59 month*” OR “0 to 36 month*” OR “6 to 36 month*” OR “0 to 24 month*” OR “24 to 59 month*” OR “24 to 59 month*” OR “0 to 2 year*” OR “0 to 5 year*” OR “2 to 5 year*”).

All identified articles were exported to EndNote version 21.5 and then imported to Covidence (Veritas Health Innovation, Melbourne, Australia. Available at www.covidence.org). Initial screening was performed by title and abstract followed by full-text access for eligible articles. Each article’s abstract and full text were then reviewed to exclude non-relevant articles. The Covidence tool automatically removed duplicates and was used to keep records of all the searching and screening processes. Two reviewers (MW and ZM) independently assessed and approved the inclusion of articles. Any discrepancies between the two reviewers were resolved through discussion. The article screening process is illustrated in the PRISMA flow diagram (Figure S1 in Supplementary File S2).

### 2.2. Eligibility Criteria and Data Extraction

Studies among under-five children reported prevalences of CIAF or any of its disaggregated forms and/or coexisting forms of undernutrition where anthropometric measurements based on the 2006 WHO Standard Growth Reference were considered. Studies conducted in SSA, published in peer-reviewed journals from 2006 onwards, employed quantitative or mixed methods, and those with outcome measures based on the CIAF (both aggregated and disaggregated forms of CIAF) and/or coexisting forms of undernutrition were considered. Only articles published in English were included (Table 1).

Studies among under-five children with congenital problems; specific population groups such as migrants or displaced children; or those with specific health conditions like Human Immunodeficiency Virus/Acquired Immune Deficiency Syndrome (HIV/AIDS), tuberculosis, anaemia, or severe disease conditions were excluded. Additionally, studies conducted outside SSA, those published before 2006, and those using growth reference tools other than the 2006 WHO growth standard were excluded. Qualitative studies, reports, commentaries, abstracts, editorials, book chapters, systematic reviews, scoping reviews, and protocols, as well as studies using conventional anthropometric indices, mid-upper arm circumference (MUAC), or body mass index (BMI), were excluded.

Data were extracted using an Excel spreadsheet (Version 2503 Build 16.0.18623.20266) by two reviewers (MW and ZM), and the extracted data were approved by two other reviewers (TN and IM). Both reviewers independently extracted estimates for various undernutrition categories and information on study characteristics. Data were extracted for prevalence estimates for the overall magnitude of undernutrition (aggregated CIAF), for various single, double, and triple forms of undernutrition, author, year of publication, country, subregion in SSA, study design, age groups of children, sample size, and sampling techniques (Table 2). Two of the authors (MW and ZM) cross-verified the data extraction for all the included studies to minimise potential biases and ensure the reliability of the extracted information.

### 2.3. Statistical Analysis

Due to high heterogeneity among the included studies, a meta-analysis was performed using a random effect model. The pooled prevalence and 95% CI were presented as a summary effect estimate for each category of undernutrition. Heterogeneity was assessed using the Higgins I^2^ (I-squared) statistic, with an I^2^ value greater than 75% indicating high heterogeneity [17]. Subgroup analysis was conducted by study covariates to identify the source of heterogeneity. Meta-regression analysis was then conducted using study characteristics as covariates to explore the sources of heterogeneity among the included studies. An asymmetry funnel plot and Egger’s test were employed to assess publication bias among the included studies. Finally, sensitivity analysis was performed by trimming low-quality studies to compare pooled estimates before and after trimming. Joanna Briggs Institute (JBI) critical appraisal tool for prevalence studies was adopted to assess the quality of the included studies. The tool was validated for its reliability [18].

## 3. Results

A total of 3898 articles were identified through database searches. After removing duplicates, 3423 studies were screened by title, and abstract and 77 studies were eligible for full-text screening. Following the full-text review, 26 studies [16,19,20,21,22,23,24,25,26,27,28,29,30,31,32,33,34,35,36,37,38,39,40,41,42,43] reporting prevalence estimates for the various categories of undernutrition were included (Figure S1 in Supplementary File S2).

### 3.1. Characteristics of the Included Study and Quality Appraisal Score

**Figure 1:**
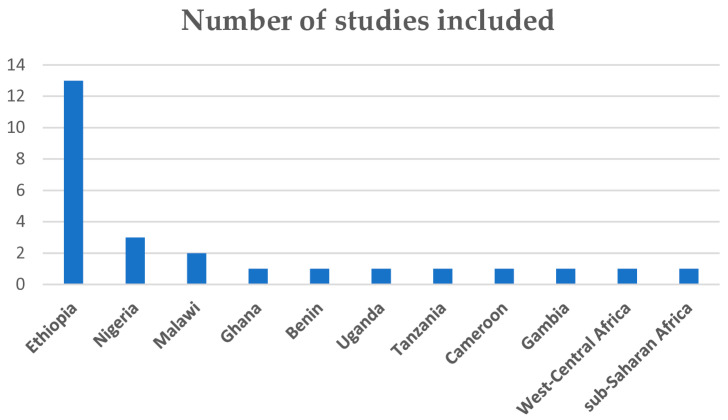
Indicates the number of included studies.

The characteristics of the included studies are summarised in Table 2. The included studies were identified from nine countries, with two additional studies conducted at subregional (West-central Africa) and SSA levels (Figure 1). About 50% of the included studies were from Ethiopia, and 19 of the included studies were based on nationally representative secondary survey data. Twenty-two studies scored 7 and above (high-quality level) on the JBI critical appraisal score, and the remaining 4 had scores of between 4 and 6 (medium-quality level). Detailed information on the quality assessment results of the included studies is provided in Table S1 of Supplementary File S3.

**Table 2:** Summarise the characteristics of the included studies.

Author (Year)	Country	Sub-Region	Age Group (M)	Study Design	Sample Size	Year of Data Collection	Sampling Techniques	Prevalence (%) by Undernutrition Categories	JBI Score
Alarape et al. (2022) [21]	Nigeria	West Africa	0 to 59	Cross-sectional	19,471	2018	Stratified-cluster sampling	Wasting only = 1.4 Underweight only = 1.8 WU = 2.4	9
Endris N et al. (2017) [29]	Ethiopia	East Africa	0 to 59	Cross-sectional	3095	2014	Stratified-cluster sampling	CIAF = 48.2 Stunting only = 18.2 Wasting only = 3.3 Underweight only = 1.3 SU = 19.4 WU = 2.8 SWU = 3.7	9
Amusa et al. (2023) [23]	Nigeria	West Africa	0 to 59	Cross-sectional	10,962	2018	Stratified-cluster sampling	CIAF = 41.3 Stunting only = 36.2 Wasting only = 6.8 Underweight only = 21.7 SU = 14.8 WU = 2.1 SWU = 3	9
Odei Obeng-Amoako et al. (2020) [39].	Uganda	East Africa	6 to 59	Cross-sectional	32,962	2015–2018	Two-stage cluster sampling	SWU = 4.96	9
Gonete AT, et al. (2021) [32]	Ethiopia	East Africa	Newborn	Cross-sectional	394	2020	Simple random sampling	SWU = 2.5	6
Bidira K et al. (2021) [27]	Ethiopia	East Africa	24 to 59	Cross-sectional	588	2019	Systematic random sampling	CIAF = 50.8 Stunting only = 22.5 Wasting only = 0.4 Underweight only = 4.9 SU = 11.1 WU = 3.7 SWU = 8.3	7
Roba A et al. (2021) [41]	Ethiopia	East Africa	6 to 59	Cross-sectional	993	2019	Simple random sampling	SWU = 5.8	9
Addo I et al. (2023) [19]	Benin	West Africa	0 to 59	Cross-sectional	13,589	2017–2018	Two-stage cluster sampling	CIAF = 14.95	9
M. Pomati, S. Nandy (2019) [37]	-	West & Central Africa	0 to 59	Cross-sectional	183,000	2008–2012	Two-stage cluster sampling	CIAF = 48 Stunting only = 21 Wasting only = 4 Underweight only = 1 SU = 14 WU = 4 SWU = 4	9
B. O. Olusanya et al. (2010) [40]	Nigeria	West Africa	0 to 3	Cross-sectional	5888	2005–2006	Two-stage cluster sampling	CIAF = 38.8 Stunting only = 21.5 Wasting only = 3.3 Underweight only = 1.2 SU = 6.1 WU = 3.4 SWU = 3.3	6
Fenta H et al. (2021) [30]	Ethiopia	East Africa	0 to 59	Cross-sectional	29,599	2000–2016	Two-stage cluster sampling	CIAF = 53.78	9
Asoba et al. (2019) [25]	Cameroon	West Africa	0 to 59	Cross-sectional	1227	2018	A multistage cluster sampling	CIAF = 32.6	9
Berra W (2020) [26]	Ethiopia	East Africa	6 to 23	Cross-sectional	525	2016	Systematic random sampling	CIAF = 21.3	5
Ziba and Kalimbiraba et al. (2018) [16]	Malawi	South Africa	0 to 59	Cross-sectional	4586	2010	Stratified-cluster sampling	CIAF = 50.6 Stunting only = 36.2 Wasting only = 1.7 Underweight only = 0.7 SU = 9.6 WU = 1.1 SWU = 1.3	9
Sahiledengle B et al. (2023) [42]	Ethiopia	East Africa	0 to 59	Cross-sectional	33,650	2000–2016	Two-stage cluster sampling	SWU = 4.7	9
Workie and Tesfaw (2021) [43]	Ethiopia	East Africa	0 to 59	Cross-sectional	9411	2016	Two-stage cluster sampling	Rural CIAF = 48.06 Urban CIAF = 33.26	9
Indris A (2021) [20]	Ethiopia	East Africa	6 to 23	Cross-sectional	245	2019	Simple random sampling	Wasting only = 6.12 Underweight only = 4.48 WU = 2.44	4
Asmare and Agmas (2022) [24]	Gambia	West Africa	0 to 59	Cross-sectional	2399	2019–2020	Two-stage cluster sampling	CIAF = 24.55 Stunting only = 4.96 Wasting only = 0.92 Underweight only = 6.54 SU = 7.13 WU = 3.58 SWU = 1.42	9
Shiferaw and Regassa (2023) [38]	Ethiopia	East Africa	0 to 59	Cross-sectional	48,782	2000–2019	Two-stage cluster sampling	CIAF = 50.71	9
Chikhungu L (2022) [28]	Malawi	South Africa	0 to 59	Cross-sectional	5127	2015	Two-stage cluster sampling	CIAF = 38.7 Stunting only = 26.8 Wasting only = 1 Underweight only = 0.4 SU = 8.8 WU = 0.8 SWU = 0.9	9
N. Fentahun et al. (2016) [31]	Ethiopia	East Africa	0 to 59	Cross-sectional	674	2015	Stratified-cluster sampling	CIAF = 46.4 Stunting only = 14.5 Wasting only = 1.5 Underweight only = 2.7 SU = 23.2 WU = 2.8 SWU = 1.8	8
Khamis et al. (2020) [34]	Tanzania	East Africa	0 to 59	Cross-sectional	6774	2010–2011	Two-stage cluster sampling	CIAF = 38.2 Stunting only = 23.2 Wasting only = 1.6 Underweight only = 0.9 SU = 9.7 WU = 1.3 SWU = 1.5	9
					8913	2014/15		CIAF = 45.9 Stunting only = 28.8 Wasting only = 1.3 Underweight only = 0.9 SU = 11.4 WU = 1.7 SWU = 1.8	
Kuwornu et al. (2022) [35]	Ghana	West Africa	6 to 59	Cross-sectional	6532	2011	Two-stage cluster sampling	CIAF = 30 Stunting only = 14.5 Wasting only = 1.5 Underweight only = 1.5 SU = 8.4 WU = 2.4 SWU = 1.6	9
					2141	2008		CIAF = 37 Stunting only = 19.5 Wasting only = 3.20 Underweight only = 0.8 SU = 8.5 WU = 3.4 SWU = 1.7	
					1608	2009/10		CIAF = 45 Stunting only = 18.4 Wasting only = 4.2 Underweight only = 0.8 SU = 7.6 WU = 9 SWU = 5.6	
Menalu et al. (2022) [36]	Ethiopia	East Africa	0 to 59	Cross-sectional	355	2019–2020	Systematic random sampling	CIAF = 15.8	4
Kassie and Workie (2019) [33]	Ethiopia	East Africa	0 to 59	Cross-sectional	8768	2016	Two-stage cluster sampling	CIAF = 45.96 Stunting only = 16.86 Wasting only = 4.22 Underweight only = 1.43 SU = 15.58 WU = 3.98 SWU = 3.89	9
Amadu I, et al. (2021) [22]	-	West Africa	0 to 59	Cross-sectional	127,487	2010–2019	Two-stage cluster sampling	SWU = 3.11	9
		East Africa							
								SWU = 2.43	
		Central Africa						SWU = 2.83	
								SWU = 0.88	
		South Africa							

CIAF = Composite Index of Anthropometric Failure (as a measure of overall undernutrition magnitude), JBI = Joanna Briggs Institute, M = month, SU = stunting-underweight, SWU = stunting-wasting-underweight, WU = wasting-underweight.

### 3.2. Pooled Prevalence of Overall Magnitude of Undernutrition

**Figure 2:**
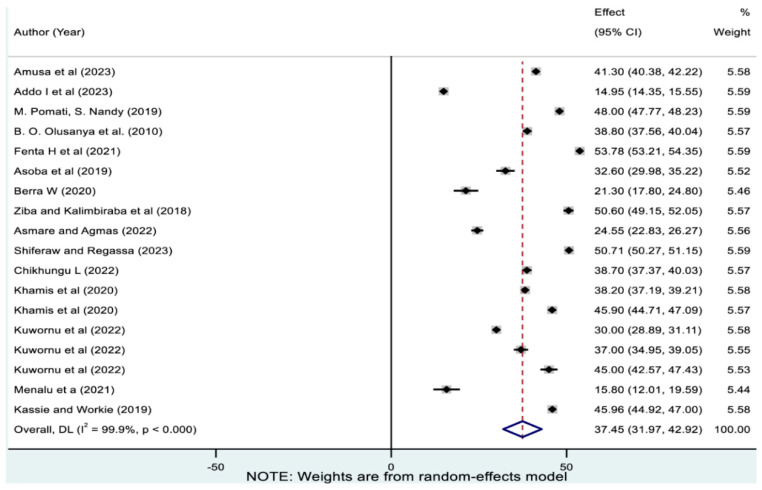
Pooled overall magnitude of undernutrition among children in sub-Saharan Africa. Note: The horizontal lines represent 95% confidence intervals, and the summary diamond indicates the effect size across all included studies. The large diamond at the bottom represents the overall pooled prevalence estimate, with its width indicating the 95% confidence interval for the combined effect.

Figure 2 showed that the random effect pooled prevalence of the overall magnitude of undernutrition (from aggregated CIAF) in SSA was 37.45% (95% CI: 31.97, 42.92). It ranged from 14.95% (95% CI: 14.35, 15.55) in Benin to 44.65% (95% CI: 32.98, 56.31) in Malawi. The highest and lowest prevalence of overall magnitude of undernutrition were reported in West-Central Africa (48% [95% CI: 47.77, 48.22]) and West Africa (33.01% [95% CI: 23.77, 42.26]), respectively (Table 3).

### 3.3. Pooled Prevalences of Single Forms of Undernutrition

Moreover, 25.25% (95% CI: 16.78, 33.72) of children in SSA experienced single forms of undernutrition, which ranged from 2.83% (95% CI: 1.94, 3.72) for wasting only to 22.32% (95% CI: 18.26, 26.39) for stunting only.

**Figure 3:**
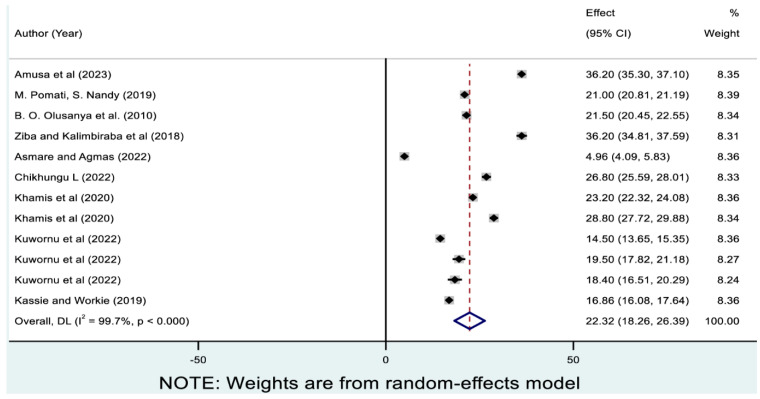
Pooled prevalence of stunting only among children in sub-Saharan Africa. Note: The horizontal lines represent 95% confidence intervals, and the summary diamond indicates the effect size across all included studies. The large diamond at the bottom represents the overall pooled prevalence estimate, with its width indicating the 95% confidence interval for the combined effect.

As presented in Figure 3, 12 studies reported the prevalence of stunting only, and the pooled prevalence was 22.32% (95% CI: 18.26, 26.39). As presented in Table 3, the highest and lowest pooled prevalences of stunting only were reported in Malawi (31.49% [95% CI: 22.28, 40.71]) and Gambia (4.96% [95% CI: 4.09, 5.83]), respectively. The pooled magnitude ranged from 19.18% (95% CI: 9.31, 29.04) in West Africa to 31.49% (95% CI: 22.28, 40.71) in South Africa.

**Figure 4:**
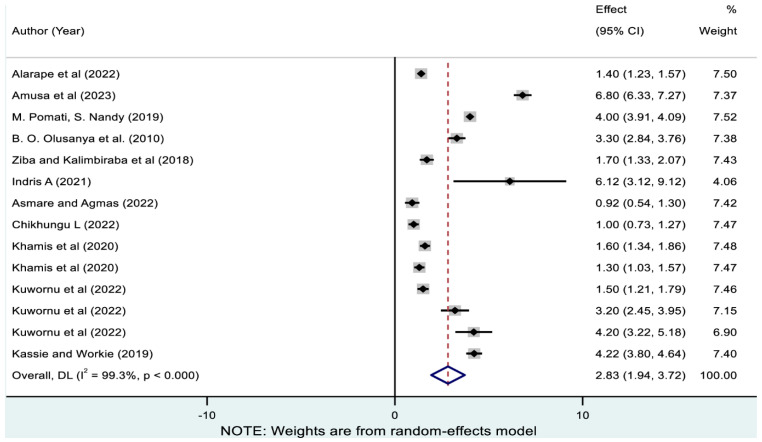
Pooled prevalence of wasting only among children in sub-Saharan Africa. Note: The horizontal lines represent 95% confidence intervals, and the summary diamond indicates the effect size across all included studies. The large diamond at the bottom represents the overall pooled prevalence estimate, with its width indicating the 95% confidence interval for the combined effect.

As demonstrated in Figure 4, the pooled magnitude of wasting only estimated from 14 studies was 2.83% (95% CI: 1.94, 3.72). As illustrated in Table 3, the highest and lowest pooled prevalence of wasting only was reported in Ethiopia (4.57% [95% CI: 3.13, 6.00]) and Gambia (0.92% [95% CI: 0.53, 1.30]), respectively. Subregionally, the prevalence was highest in West-Central Africa (4% [95% CI: 3.91, 4.09]) and lowest in South Africa (1.34% [95% CI: 0.65, 2.02]).

**Figure 5:**
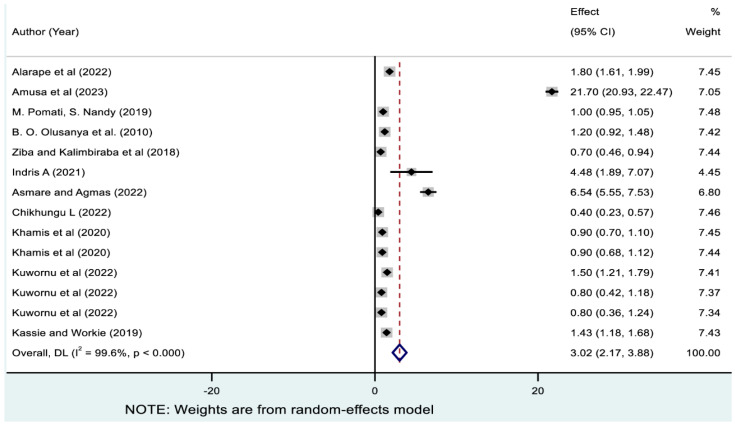
Pooled prevalence of underweight only among children in sub-Saharan Africa. Note: The horizontal lines represent 95% confidence intervals, and the summary diamond indicates the effect size across all included studies. The large diamond at the bottom represents the overall pooled prevalence estimate, with its width indicating the 95% confidence interval for the combined effect.

As indicated in Figure 5, the pooled prevalence of underweight only estimated from 14 studies was 3.02% (95% CI: 2.17, 3.88). As revealed in Table 3, the highest prevalence of underweight only was reported in Nigeria (8.21% [95%CI: 1.79, 14.63]), and the lowest was in Malawi (0.54% [95% CI: 0.24, 0.83]). Subregionally, West Africa exhibited the highest pooled prevalence of underweight only (4.88% [95% CI: 2.11, 7.65]), while South Africa exhibited the lowest (0.54% [95% CI: 0.24, 0.83]).

### 3.4. Pooled Prevalences of Coexisting Forms of Undernutrition

Furthermore, 10% (95% CI: 8.02, 11.98) of children in SSA experienced multiple forms of undernutrition. Coexisting forms of undernutrition ranged from 2.82% (95% CI: 2.19, 3.44) in the triple form to 10.15% (95% CI: 8.17, 12.13) in the double form (stunting-underweight).

**Figure 6:**
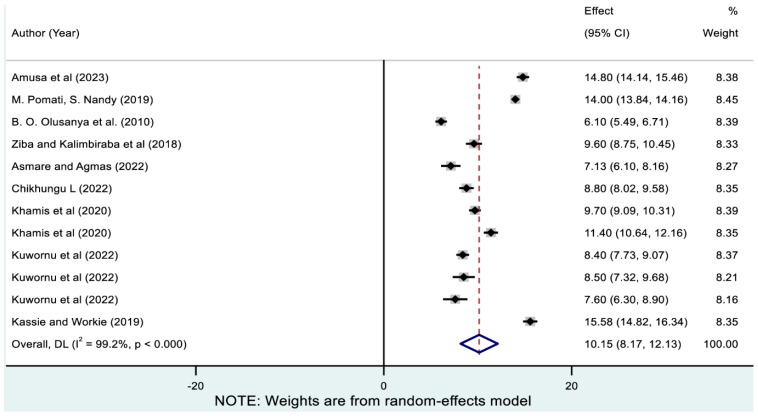
Pooled prevalence of coexistence of stunting with underweight among children in sub-Saharan Africa. Note: The horizontal lines represent 95% confidence intervals, and the summary diamond indicates the effect size across all included studies. The large diamond at the bottom represents the overall pooled prevalence estimate, with its width indicating the 95% confidence interval for the combined effect.

As indicated in Figure 6, 12 studies reported the co-existence of stunting with underweight, and the pooled prevalence was 10.15% (95% CI: 8.17, 12.13). As reported in Table 3, the prevalence of stunting-underweight ranged from 7.13% (95% CI: 6.10, 8.16) in Gambia to 15.58% (95% CI: 14.82, 16.34) in Ethiopia. The lowest and highest prevalence of coexisting stunting with underweight was observed in West Africa (8.76% [95% CI: 5.76, 11.77]) and West-Central Africa (14% [95% CI: 13.84, 14.16]), respectively.

**Figure 7:**
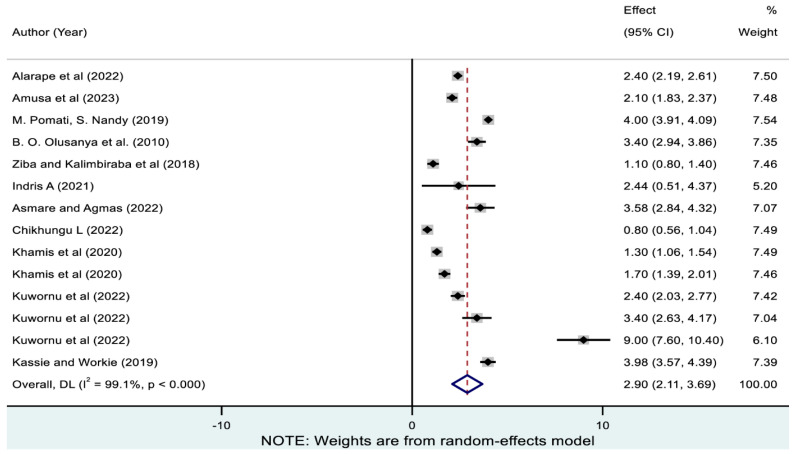
Pooled prevalence of coexistence of wasting with underweight among children in sub-Saharan Africa. Note: The horizontal lines represent 95% confidence intervals, and the summary diamond indicates the effect size across all included studies. The large diamond at the bottom represents the overall pooled prevalence estimate, with its width indicating the 95% confidence interval for the combined effect.

As presented in Figure 7, the pooled magnitude of coexisting wasting with underweight calculated from 14 studies was 2.90% (95% CI: 2.11, 3.69). As demonstrated in Table 3, co-existence of wasting with underweight ranged from 0.94% (95% CI: 0.64, 1.23) in Malawi to 4.84% (95% CI: 2.06, 7.62) in Ghana. Subregionally, the co-existence of wasting with underweight varied from 0.94% (95% CI: 0.64, 1.23) in South Africa to 4% (95% CI: 3.91, 4.09) in West-Central Africa.

**Figure 8:**
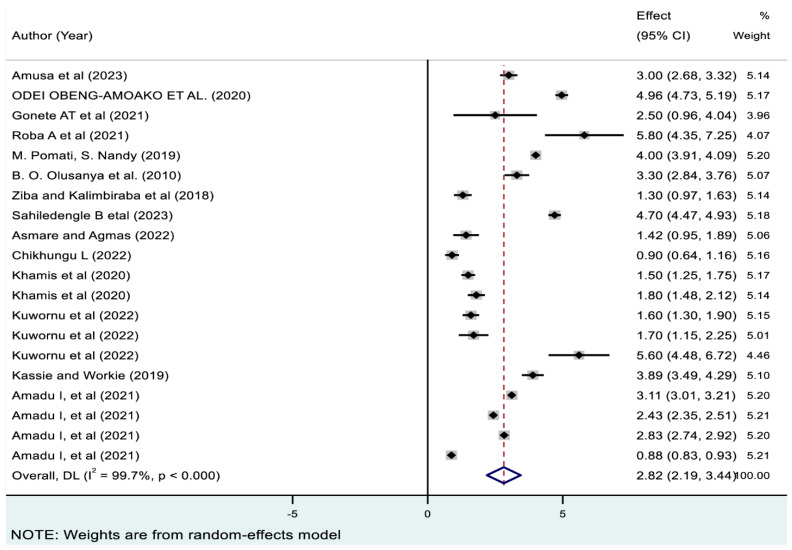
Pooled prevalence of triple coexistence of stunting-wasting-underweight among children in sub-Saharan Africa. Note: The horizontal lines represent 95% confidence intervals, and the summary diamond indicates the effect size across all included studies. The large diamond at the bottom represents the overall pooled prevalence estimate, with its width indicating the 95% confidence interval for the combined effect.

As presented in Figure 8, the pooled prevalence of triple forms of undernutrition among 20 included studies was 2.82% (95% CI: 2.19, 3.44). As depicted in Table 3, it was lowest in Malawi (1.09 [95% CI: 0.7, 1.48]) and highest in Uganda (4.96% [95% CI: 4.72, 5.19]). Subregionally, this prevalence varied from 0.98% (95% CI: 0.77, 1.19) in South Africa to 4.00% (95% CI: 3.91, 4.00) in West-central Africa.

### 3.5. Meta-Regression Analysis

Meta-regression analysis was conducted using study covariates to explore the sources of high levels of heterogeneity observed among the various categories of undernutrition. Study covariates such as year of study, sample size, and subregions in SSA were significant sources of heterogeneity among studies included for estimating the overall magnitude of undernutrition (aggregated CIAF). Subregions in SSA and sample size were significant sources of heterogeneity among studies included to obtain the pooled prevalence of the triple forms of undernutrition (coexistence of stunting-wasting-underweight) (*p*-value ≤ 0.05). In contrast, none of the study covariates explained the heterogeneity observed among other undernutrition categories (*p* > 0.05). Detailed meta-regression results are presented in Table S2 of Supplementary File S4.

Moreover, this study stratified the pooled overall magnitude of undernutrition (aggregated CIAF) and triple forms of undernutrition by gender and place of residence. Four studies reported aggregated CIAF prevalence by gender. The pooled magnitude was 31.74% (95% CI: 17.39, 46.09) for male and 37.38% (95% CI: 19.18, 55.57) for female children. The meta-regression analysis showed non-significant gender variation in the pooled prevalence of overall magnitude of undernutrition (aggregated CIAF). Three studies reported prevalence for triple undernutrition by gender, where the pooled prevalence among male children (6.03% [95% CI: 3.50, 8.50]) almost doubled the female prevalence (3.07% [95% CI: 1.69, 4.44]). However, the difference was not statistically significant (*p*-value > 0.05). Furthermore, five studies reported the prevalence of aggregated CIAF for rural children. The random effect pooled prevalence was 49.70% (95% CI: 45.95, 53.45). Two studies reported the prevalence of aggregated CIAF among urban children, and the pooled prevalence was 33.52% (95% CI: 32.43, 34.60). The meta-regression analysis showed a statistically significant rural-urban difference in aggregated CIAF, where being rural has a 16 times higher risk of experiencing at least one form of undernutrition compared with their urban counterpart (*p*-value ≤ 0.05).

For triple undernutrition, five studies reported prevalence among rural children, resulting in a pooled prevalence of 4.04% (95% CI: 2.92, 5.15). Likewise, two studies reported prevalence among urban children, with a pooled prevalence of 2.07% (95% CI: 1.31, 2.83) (Table 4).

The meta-regression analysis did not find a significant rural-urban difference in the triple forms of undernutrition. There were insufficient studies to explore gender differences and residential variation in other undernutrition categories (stunting only, wasting only, underweight only, stunting-underweight, and wasting-underweight).

### 3.6. Publication Bias and Sensitivity Analysis

Publication bias was assessed both subjectively and objectively for each undernutrition category. The asymmetry funnel plot showed a substantial publication bias among studies included to estimate aggregated CIAF, stunting only, wasting only, and the triple forms (stunting-wasting-underweight). Conversely, the funnel plot showed no publication bias among studies estimating underweight only, coexistence of stunting with underweight, and co-occurrence of wasting with underweight (Figures S2–S8 of Supplementary File S5).

Objectively, Egger’s test found no statistically significant publication bias for aggregated CIAF, stunting only, wasting only, underweight only, wasting with underweight, and triple undernutrition (*p*-value > 0.05). However, there was statistically significant publication bias among studies estimating the stunting-underweight category (*p*-value ≤ 0.05) (Table 5). Finally, sensitivity analysis was performed by excluding low-quality studies (studies with a quality score less than 7). After trimming low-quality studies, the pooled prevalence of various undernutrition categories remained unchanged (Table S3 of Supplementary File S6).

## 4. Discussion

This is the first systematic review and meta-analysis to provide a comprehensive overview of current evidence on the prevalence of various forms of undernutrition using the CIAF approach and to identify the most prevalent undernutrition patterns. It is also the first to quantify the pooled prevalence for the overall magnitude of undernutrition and prevalences for the various disaggregated patterns of undernutrition. Unlike previous studies [44,45], which relied on conventional undernutrition measurements and were limited to certain undernutrition categories, this systematic review and meta-analysis has a broader scope and employed the more comprehensive undernutrition measurement approach (CIAF). The present study revealed that in SSA, nearly two in five children are undernourished, and about one-third of these undernourished children experienced coexisting forms of undernutrition.

This study also identified stunting only and the co-existence of stunting with underweight as the two most prevalent undernutrition patterns, followed by underweight only and the co-existence of wasting with underweight. This finding is consistent with a few other previous studies conducted elsewhere [10,21,46,47]. This supports the hypothesis that undernutrition manifestation among children is not limited to the conventional forms—stunting, wasting and underweight; instead, a substantial proportion of children are affected by the combinations of various forms of undernutrition.

This study also found that about 37.45% of children in SSA experienced at least one form of undernutrition. This prevalence estimate is higher than those obtained using conventional undernutrition measurements for the SSA region [48]. However, it is lower than other country-specific reports [49,50,51]. This indicates that the overall population-level magnitude of undernutrition, calculated from aggregated CIAF, is significantly higher than the prevalence estimates for stunting, wasting, and underweight independently. For the past three decades, one of the three conventional undernutrition measurements has been used as a policy-relevant undernutrition indicator. However, since these conventional measures each individually miss a large number of children and cannot distinguish those with coexisting forms of undernutrition, their policy implications are limited. This highlights that many undernourished children could have benefitted from policy and programme considerations if the composite undernutrition measurement had been used to redefine the nutritional status of children. Therefore, it is advisable to customise current child nutrition policy, programme design, and resource allocation based on the more comprehensive undernutrition assessment technique (CIAF).

Furthermore, evidence from key organisations dedicated to combating undernutrition indicates that a significant number of under-five children are experiencing coexisting forms of undernutrition. These children exhibit distinct clinical manifestations and presentations compared with those experiencing a single form of undernutrition [16,52]. Reports from these organisations also emphasise that various forms of undernutrition are interrelated (one form can lead to another) and share common risk factors [53].

Similarly, this study found high rates of coexisting forms of undernutrition. The pooled prevalence of coexisting stunting with underweight (10.15%) in this study is higher than CIAF-based reports from Brazil [54] and China [55] but lower than findings from Bangladesh [51] and India [56]. Additionally, a significant percentage (2.90%) of children were affected by coexisting wasting with underweight. This finding aligns with the reports from China [55] and Pakistan [44], although a study from Bangladesh reported a higher magnitude of this coexisting form [51]. Moreover, the pooled prevalence of triple forms of undernutrition (2.82%) in this study is consistent with the 2019 global nutrition report for the Africa region [57]. However, this estimate is lower than the one from another primary study conducted in Bangladesh [51]. Differences in the prevalence of various coexisting forms of undernutrition across regions could be attributed to the variations in nutritional beliefs, practices, and inequalities in access to quality healthcare and health policies [22]. Consequently, identifying, targeting, and addressing the most prevalent coexisting undernutrition patterns within a particular geographic region would aid in alleviating the burdens associated with these forms of undernourishment.

This study also found high rates of various single forms of undernutrition. The pooled prevalence of stunting only (22.32%) in SSA is higher than the findings from India [10], Bangladesh [51], Yemen [52], and Indonesia [49]. The pooled magnitude of wasting only (2.83%) is in agreement with findings from India [10], Indonesia [49], and Bangladesh [51] but lower compared to the report from Yemen [52]. Moreover, the pooled prevalence of underweight only in SSA was 3.02%, which is consistent with reports from India and Bangladesh [10,51]. This finding is lower than the report from Indonesia [49]. This variation in the prevalence of several forms of undernutrition among children could be linked to the variation in socioeconomic status, food insecurity, and political instability [52].

In this study, there was no significant gender variation in CIAF and triple forms of undernutrition, which is consistent with previous studies [44,46,58]. However, other studies reported a significant gender variation in various undernutrition forms [41,59,60]. The absence of significant gender difference in this study might be due to the limited number of the included studies or may reflect an insufficient sample due to stratification. Yet, there is no known biological reason for gender variation in undernutrition prevalence. However, existing studies justified this, as male children are physically more active and spend more energy compared with their female counterparts, which could lead to depletion of available body energy and affect child growth [45]. This study identified a significant rural-urban difference in aggregated CIAF prevalence, with rural children more likely to experience it compared to urban children. This finding is consistent with previous studies conducted elsewhere [49,61]. The elevated prevalence of overall magnitude of undernutrition in rural children might stem from factors such as limited access to universal primary education, inadequate nutritional awareness, restricted access to healthcare services, and inadequate food access [62].

In this review, there was a non-significant rural-urban difference in triple forms of undernutrition, which is supported by previous studies conducted in Pakistan [44]. Rural-urban differences for other undernutrition categories were not investigated in this study due to the scarce studies. The population distribution, socioeconomic status, and healthcare practice significantly vary by place of residence, particularly in developing countries [50,63]. Thus, it is advisable to comprehensively investigate the rural-urban distribution of different forms of undernutrition among children. This examination would aid in healthcare planning, policy formulation, and programme development to alleviate existing residential inequalities of undernutrition in SSA.

Based on this review, we recommend adopting the CIAF approach, as it is crucial for stratifying child risk based on the type of undernutrition. For instance, in conventional undernutrition classification, children with stunting only, stunting-underweight, and stunting-wasting-underweight are all labelled as “stunted”. In contrast, the CIAF approach distinguishes between these categories and highlights that children with stunting-underweight or stunting-wasting-underweight face higher risks of illness and mortality [64]. This distinction allows health programmes to go beyond a one-size-fits-all approach and design a more inclusive and risk-sensitive strategy, ensuring that the most vulnerable children are identified and prioritised in nutrition policies, programmes, and treatment efforts.

### Strengths and Limitations of the Study

The key strength of this study is that it is the first systematic review and meta-analysis to comprehensively explore current evidence on different patterns of undernutrition using the CIAF among under-five children in the SSA context. Unlike previously published studies, we estimated pooled prevalence for the overall magnitude of undernutrition (aggregated CIAF) and identified and quantified pooled magnitudes for six disaggregated patterns of undernutrition (stunting only, wasting only, underweight only, stunting-underweight, wasting-underweight, and stunting-wasting-underweight). Another important strength of this study lies in its utilisation of a consistent undernutrition measurement standard, adhering to the 2006 WHO multi-growth reference standard. This enhances the generalisability and applicability of the findings of this study elsewhere.

Nevertheless, this study has certain limitations. First, the included studies have restricted geographic coverage, and some are confined to limited undernutrition patterns (limited subgroups of CIAF). As a result, the findings of this study might not accurately represent the real undernutrition situation in SSA. Additionally, we were unable to stratify and assess variation in various forms of undernutrition by key demographic characteristics such as age, gender, and geographical region due to an insufficient number of studies. However, undernutrition manifestations can significantly vary by these characteristics. Therefore, a more inclusive study using a representative dataset is still needed to further explore the different forms of undernutrition and assess its variation by these population characteristics across SSA countries.

Finally, the pooled prevalence obtained in this study likely underestimates the true burden of undernutrition, as high-risk groups, such as children with congenital conditions, severe illnesses (e.g., HIV/AIDS, tuberculosis, and anaemia), and marginalised populations like migrants, were excluded from the analysis. These groups often face higher nutritional risk due to biological vulnerability and limited access to care.

## 5. Conclusions

In conclusion, the present study found a worryingly high overall magnitude of undernutrition among under-five children with a significant rate of co-existence of various forms of undernutrition. These coexisting forms of undernutrition might be significantly linked to other child health conditions, potentially leading to elevated levels of under-five mortality and other related health complications from preventable causes. This study also identified subregions and countries in SSA that are highly affected by various single, double, and triple forms of undernutrition. This underscores the urgency of implementing impactful region-specific policies targeting the most prevalent patterns of undernutrition.

Policies and interventions targeted at undernutrition reduction need to be tailored by considering the actual magnitude of undernutrition and the coexisting forms. We strongly suggest the adoption of the CIAF in both community- and facility-based child nutrition screening programmes, treatment approaches, and routine child growth assessment reports. Including data on multiple forms of undernutrition should become standard in child nutrition surveillance systems, rather than relying on standalone indicators. Specifically, WHO and UNICEF should support the development of integrated screening protocols for identifying multiple anthropometric failures and promote multi-indicator nutritional strategies to achieve the SDG’s goal of eliminating all forms of child undernutrition by 2030. Finally, global organisations should adopt CIAF thresholds to classify population-level nutritional risk and inform funding priorities.

## Figures and Tables

**Table 1:** Categorisation of the composite index of anthropometric failure.

CIAF Categories	Wasted	Stunted	Underweight
No failure	No	No	No
Stunting only	No	Yes	No
Wasting only	Yes	No	No
Underweight only	No	No	Yes
Stunted and underweight	No	Yes	Yes
Wasted and underweight	Yes	No	Yes
Wasted, stunted, and underweight	Yes	Yes	Yes

**Table 3:** Subgroup analysis on the pooled prevalences of various forms of undernutrition.

Categories	Overall Magnitude of Undernutrition		Single Forms of Undernutrition						Coexisting Forms of Undernutrition					
	CIAF		Stunting Only		Wasting Only		Underweight Only		SU		WU		SWU	
	N	Prevalence (%) 95%CI	N	Prevalence (%)	N	Prevalence (%)	N	Prevalence (%)	N	Prevalence (%)	N	Prevalence (%)	N	Prevalence (%)
Overall	18	37.45 (31.97–42.92)	11	22.32 (18.26–26.39)	14	2.83 (1.94–3.72)	14	3.02 (2.17–3.88)	12	10.15 (8.17–12.13)	14	2.90 (2.11–3.69)	20	2.82 (2.19–3.44)
Country														
Ethiopia	5	38.15 (32.62–43.66)	1	16.86 (16.07–17.64)	2	4.57 (3.13–6.00)	2	2.67 (0.27–5.61)	1	15.58 (14.82–16.34)	2	3.51 (2.12–4.90)	4	4.28 (3.49–5.06)
Ghana	3	37.28 (28.67–45.89)	3	17.4 (13.93–20.87)	3	2.92 (1.25–4.59)	3	1.05 (0.55–1.55)	3	8.28 (7.75–8.82)	3	4.84 (2.06–7.62)	3	2.85 (1.27–4.42)
Tanzania	2	42.04 (34.49–49.58)	2	25.99 (20.50–31.48)	2	1.45 (1.16–1.75)	2	0.9 (0.75–1.05)	2	10.54 (8.87–12.20)	2	1.49 (1.10–1.88)	2	1.63 (1.34–1.93)
Nigeria	2	40.09 (37.64–42.53)	2	28.85 (14.45–43.26)	3	3.83 (0.65–7.00)	3	8.21 (1.79–14.63)	2	10.45 (1.92–18.97)	3	2.6 (2.02–3.17)	2	3.10 (2.82–3.38)
Malawi	2	44.65 (32.98–56.31)	2	31.49 (22.28–40.71)	2	1.34 (0.65–2.02)	2	0.54 (0.24–0.83)	2	9.18 (8.40–9.96)	2	0.94 (0.64–1.23)	2	1.09 (0.7–1.48)
Gambia	1	24.55 (22.83–26.27)	1	4.96 (4.09–5.82)	1	0.92 (0.53–1.30)	1	6.54 (5.55–7.52)	1	7.13 (6.10–8.16)	1	3.58 (2.83–4.32)	1	1.42 (0.94–1.89)
Uganda	-	-	-	-	-	-	-	-		-	-	-	1	4.96 (4.72–5.19)
Benin	1	14.95 (14.35–15.55)	-	-	-	-	-	-	-	-		-	-	-
Cameroon	1	33.6 (29.98, 35.22)	-	-	-	-	-	-	-	-		-	-	-
Overall, DL	I^2^ = 99.9%, *p* < 0.000		I^2^ = 99.7%, *p* < 0.000		I^2^ = 98.4%, *p* < 0.000		I^2^ = 99.6%, *p* < 0.000		I^2^ = 98.5%, *p* < 0.000		I^2^ = 97.2%, *p* < 0.000		I^2^ = 98.8%, *p* < 0.000	
Subregion														
West Africa	8	33.01 (23.77–42.26)	6	19.18 (9.31–29.04)	7	3.03 (1.68–4.38)	7	4.88 (2.11–7.65)	6	8.76 (5.76–11.77)	7	3.46 (2.75–4.17)	7	2.72 (2.05–3.38)
West-central Africa	1	48 (47.77–48.22)	1	21 (20.81–21.18)	1	4 (3.91–4.09)	1	1 (0.95–1.04)	1	14 (13.84–14.15)	1	4 (3.91–4.09)	1	4.00 (3.91–4.00)
South Africa	2	44.65 (32.98–56.31)	2	31.49 (22.28–40.71)	2	1.34 (0.65–2.02)	2	0.54 (0.24–0.83)	2	9.18 (8.40–9.96)	2	0.94 (0.64–1.23)	3	0.98 (0.77–1.19)
East Africa	7	39.20 (34.06–44.34)	3	22.95 (16.30–29.59)	4	2.84 (1.43–4.26)	4	1.14 (0.76–1.53)	3	12.22 (8.79–15.65)	4	2.34 (1.06–3.61)	8	3.41 (2.40–4.42)
Central Africa										-	-	-	1	2.83 (2.74–2.92)
Overall, DL	I^2^ = 99.9%, *p* < 0.000		I^2^ = 99.7%, *p* < 0.000		I^2^ = 99.3%, *p* < 0.000		I^2^ = 99.6%, *p* < 0.000		I^2^ = 99.2%, *p* < 0.000		I^2^ = 99.1%, *p* < 0.000		I^2^ = 99.7%, *p* < 0.000	
Age group														
0 to 3	1	38.8 (37.55–40.04)	1	21.5 (20.45–22.54)	1	3.3 (2.84–3.75)	1	1.2 (0.92–1.47)	1	6.1 (5.48–6.71)	1	3.4 (2.93–3.86)	1	3.3 (2.84–3.75)
6 to 23	1	21.3 (17.79–24.8)		NA	1	6.12 (3.11, 9.12)	1	4.48 (1.89–7.07)			1	2.44 (0.50–4.37)		-
6 to 59	3	33.45 (32.55–34.36)	3	15.92 (15.22–16.63)	3	1.9 (1.64–2.17)	3	1.13 (0.93–1.34)	3	8.28 (7.75–8.81)	3	2.94 (2.61–3.26)	5	3.58 (3.41–3.75)
0 to 59	13	45.24 (45.07–45.41)	8	21.33 (21.16–21.50)	9	2.97 (2.91–3.04)	9	1.06 (1.02–1.10)	8	13.37 (13.23–13.51)	9	3.01 (2.94–3.08)	13	2.15 (2.11–2.18)
0												-	1	2.5 (0.96, 4.04)
Overall DL	I^2^ = 99.9%, *p* < 0.000		I^2^ = 99.7%, *p* < 0.000		I^2^ = 99.3%, *p* < 0.000		I^2^ = 99.6%, *p* < 0.000		I^2^ = 99.2%, *p* < 0.000		I^2^ = 99.1%, *p* < 0.000			
Quality level														
Medium	3	25.37 (9.60–41.15)	1	21.5 (20.45–22.55)	2	4.30 (1.66–6.95)	2	2.58 (−0.60–5.75)	1	6.1 (5.48–6.71)	2	3.35 (2.90–3.80)	2	3.24 (2.80–3.67)
High	15	39.82 (33.82–45.83)	11	22.40 (17.98–26.82)	12	2.64 (1.67–3.60)	12	3.11 (2.17–4.05)	11	10.52 (8.71–12.33)	12	2.88 (2.02–3.74)	18	2.80 (2.15–3.46)
Overall, DL	I^2^ = 99.9%, *p* < 0.000		I^2^ = 99.7%, *p* < 0.000		I^2^ = 99.3%, *p* < 0.000		I^2^ = 99.6%, *p* < 0.000		I^2^ = 99.2%, *p* < 0.000		I^2^ = 99.1%, *p* < 0.000		I^2^ = 99.7%, *p* < 0.000	
Data sources														
Primary	4	27.23 (16.94–37.52)	1	21.5 (20.45–22.54)	2	4.30 (1.66–6.95)	2	2.58 (−0.60–5.75)	1	6.01 (5.48–6.71)	2	3.35 (2.90–3.80)	3	3.83 (2.22–5.43)
Secondary	14	40.33 (34.12–46.55)	11	22.4 (17.98–26.82)	12	2.64 (1.67–3.60)	12	3.11 (2.17–4.05)	11	10.52 (8.71–12.33)	12	2.88 (2.02–3.74)	17	2.66 (1.99–3.34)
Overall, DL	I^2^ = 99.9%, *p* < 0.000		I^2^ = 99.7%, *p* < 0.000		I^2^ = 99.3%, *p* < 0.000		I^2^ = 99.6%, *p* < 0.000		I^2^ = 99.2%, *p* < 0.000		I^2^ = 99.1%, *p* < 0.000		I^2^ = 99.7%, *p* < 0.000	

CIAF = Composite Index of Anthropometric Failure, N = number of studies, SU = stunting-underweight, SWU = stunting-wasting-underweight, WU = wasting-underweight. Secondary data sources are previously collected datasets, like DHS and multiple indicator cluster surveys (MICS), used for new research purposes.

**Table 4:** Meta-regression output for the aggregated composite index of anthropometric failure and triple forms of undernutrition.

Covariates		Pooled Prevalence(95%CI)		Meta-Regression Coefficient		Std. Err		*p*-Value	
		CIAF(%)	SWU(%)	CIAF	SWU	CIAF	SWU	CIAF	SWU
**Residence**	Urban	33.52 (32.43, 34.60)	2.07 (1.31, 2.83)	-	-	-	-	-	-
	Rural	49.70 (45.95, 53.45)	4.04 (2.92, 5.15)	16.37 (10.16, 22.60)	2.085 (−2.2, 7.57)	2.42	1.67	0.001	0.26
**Sex**	Female	37.38 (19.18, 55.57)	3.07 (1.69, 4.44)	-	-	-	-	-	-
	Male	31.74% (17.39, 46.09)	6.03 (3.50, 8.50)	−5.68 (−35.22, 23.85)	2.81 (−1.79, 7.43)	12.07	1.66	0.65	0.16

CIAF = Composite Index of Anthropometric Failure, SWU = stunting-wasting-underweight.

**Table 5:** Egger’s test for the assessment of publication bias.

		Coefficient	se	t	*p*-Value	95%CI
**CIAF**	Slope	49.10	3.46	14.20	0.00	41.77, 56.43
	Bias	−16.55	9.76	−1.69	0.11	−37.26, 4.16
**Stunting only**	Slope	20.48	2.21	9.26	0.000	15.55, 25.41
	Bias	2.85	7.83	0.36	0.724	−14.60, 20.29
**Wasting only**	Slope	3.42	0.64	5.38	0.000	2.04, 4.81
	Bias	−5.19	5.23	−0.99	0.34	−16.59, 6.20
**Underweight only**	Slope	0.67	0.39	1.69	0.12	−0.192, 1.52
	Bias	8.04	5.30	1.52	0.16	−3.52, 19.59
**SU**	Slope	14.89	0.86	17.23	0.000	12.97, 16.82
	Bias	−12.19	3.73	−3.27	0.008	−20.50, 3.88
**WU**	Slope	3.59	0.54	6.62	0.00	2.41, 4.77
	Bias	−5.82	4.38	−1.33	0.21	−15.37, 3.72
**SWU**	Slope	1.81	0.41	4.38	0.00	0.944, 2.68
	Bias	7.32	5.76	1.27	0.22	−4.78, 19.42

CIAF = Composite Index of Anthropometric Failure, SU = stunting-underweight, SWU = stunting-wasting-underweight, WU = wasting-underweight.

## Supplementary Materials

The following supporting information can be downloaded at: https://www.mdpi.com/article/10.3390/nu17111818/s1, Additional File S1. Database searches for Systematic Review and meta-analysis; Figure S1. Flow diagram of the included studies; Figure S2. Funnel plot for assessing the publication bias of studies included for CIAF; Figure S3. Funnel plot for assessing the publication bias of studies included for stunting only; Figure S4. Funnel plot for assessing the publication bias of studies included for wasting only; Figure S5. Funnel plot for assessing the publication bias of studies included for underweight only; Figure S6. Funnel plot for assessing the publication bias of studies included for stunting with underweight; Figure S7. Funnel plot for assessing the publication bias of studies included for concurrent wasting-underweight; Figure S8. Funnel plot for assessing the publication bias of studies included for triple coexistence of undernutrition; Table S1: Contain tables for quality appraisal scores result; Table S2: Showing the meta-regression analysis for the various categories of undernutrition by study covariates; Table S3: Sensitivity analysis after removing studies with low quality appraisal score.

## References

[1] Vassilakou T. (2021). Childhood Malnutrition: Time for Action. Children.

[2] Jeong J., Kim R., Subramanian S. (2019). Multiple anthropometric failures and early child development in 34 low-and middle-income countries. J. Glob. Health Sci..

[3] Svedberg P. (2024). The Composite Index of Anthropometric Failure: Empirical Applications. Ann. Pediatr. Child Health.

[4] Agho K.E., Akombi B.J., Ferdous A.J., Mbugua I., Kamara J.K. (2019). Childhood undernutrition in three disadvantaged East African Districts: A multinomial analysis. BMC Pediatr..

[5] Akombi B.J., Agho K.E., Hall J.J., Merom D., Astell-Burt T., Renzaho A.M. (2017). Stunting and severe stunting among children under-5 years in Nigeria: A multilevel analysis. BMC Pediatr..

[6] Boah M., Azupogo F., Amporfro D.A., Abada L.A. (2019). The epidemiology of undernutrition and its determinants in children under five years in Ghana. PLoS ONE.

[7] Chawla S., Gupta V., Singh A., Grover K., Panika R.K., Kaushal P., Kumar A. (2020). Undernutrition and associated factors among children 1–5 years of age in rural area of Haryana, India: A community based cross-sectional study. J. Fam. Med. Prim. Care.

[8] Wells J.C., Briend A., Boyd E.M., Berkely J.A., Hall A., Isanaka S., Webb P., Khara T., Dolan C. (2019). Beyond wasted and stunted—A major shift to fight child undernutrition. Lancet Child Adolesc. Health.

[9] Kundan I., Nair R., Kulkarni S., Deshpande A., Jotkar R., Phadke M. (2021). Assessment, outcomes and implications of multiple anthropometric deficits in children. BMJ Nutr. Prev. Health.

[10] Akhade K.S. (2017). Measuring malnutrition: Needs a comprehensive indicator. Int. J. Community Med. Public Health.

[11] Sachdev H. (1995). Assessing child malnutrition: Some basic issues. Bull. Nutr. Found. India.

[12] Svedberg P. (2000). Poverty and Undernutrition: Theory, Measurement, and Policy.

[13] Nandy S., Irving M., Gordon D., Subramanian S.V., Smith G.D. (2005). Poverty, child undernutrition and morbidity: New evidence from India. Bull. World Health Organ..

[14] Nandy S., Svedberg P. (2012). The Composite Index of Anthropometric Failure (CIAF): An alternative indicator for malnutrition in young children. Handbook of Anthropometry: Physical Measures of Human Form in Health and Disease.

[15] United Nations (2015). Transforming Our World: The 2030 Agenda for Sustainable Development.

[16] Ziba M., Kalimbira A.A., Kalumikiza Z. (2018). Estimated burden of aggregate anthropometric failure among Malawian children. S. Afr. J. Clin. Nutr..

[17] Schulzke S., Patole S. (2021). Assessing and Exploring Heterogeneity. Principles and Practice of Systematic Reviews and Meta-Analysis.

[18] Khalil H., Bennett M., Godfrey C., McInerney P., Munn Z., Peters M. (2020). Evaluation of the JBI scoping reviews methodology by current users. JBI Evid. Implement..

[19] Addo I.Y., Boadu E.F., Osei Bonsu E., Boadi C., Dadzie F.A. (2023). Prevalence and factors associated with undernutrition among children under the age of five years in Benin. PLoS ONE.

[20] Indris A., Shaleka D., Ashenafi M. (2021). Child nutritional status, mothers’ nutritional knowledge and practice and Household food security status in Tehuledere Woreda, South Wollo, Ethiopia. SIN Ethiop. J. Sci..

[21] Alarape K., Yusuf O., Akinyemi J., Samuel F., Morhason-Bello I., Salami K., Obisesan O., Ilori T., Aderinto A., Alada A. (2022). Prevalence and patterns of anthropometric failure among under-five children in Nigeria: Evidence from the National nutrition and health survey, 2018. Afr. J. Reprod. Health.

[22] Amadu I., Seidu A.-A., Duku E., Frimpong J.B., Jnr J.E.H., Aboagye R.G., Ampah B., Adu C., Ahinkorah B.O. (2021). Risk factors associated with the coexistence of stunting, underweight, and wasting in children under 5 from 31 sub-Saharan African countries. BMJ Open.

[23] Amusa L.B., Yahya W.B., Bengesai A.V. (2023). Spatial variations and determinants of malnutrition among under-five children in Nigeria: A population-based cross-sectional study. PLoS ONE.

[24] Asmare A.A., Agmas Y.A. (2022). Determinants of coexistence of stunting, wasting, and underweight among children under five years in the Gambia; evidence from 2019/20 Gambian demographic health survey: Application of multivariate binary logistic regression model. BMC Public Health.

[25] Asoba G.N., Sumbele I.U.N., Anchang-Kimbi J.K., Metuge S., Teh R.N. (2019). Influence of infant feeding practices on the occurrence of malnutrition, malaria and anaemia in children≤ 5 years in the Mount Cameroon area: A cross sectional study. PLoS ONE.

[26] Berra W.G. (2020). Household food insecurity predicts childhood undernutrition: A cross-sectional study in West Oromia (Ethiopia). J. Environ. Public Health.

[27] Bidira K., Tamiru D., Belachew T. (2021). Anthropometric failures and its associated factors among preschool-aged children in a rural community in southwest Ethiopia. PLoS ONE.

[28] Chikhungu L.C. (2022). Trends and patterns of stunted only and stunted underweight children in Malawi: A confirmation for child nutrition practitioners to continue focusing on stunting. Malawi Med. J..

[29] Endris N., Asefa H., Dube L. (2017). Prevalence of malnutrition and associated factors among children in rural Ethiopia. BioMed Res. Int..

[30] Fenta H.M., Zewotir T., Muluneh E.K. (2021). Disparities in childhood composite index of anthropometric failure prevalence and determinants across Ethiopian administrative zones. PLoS ONE.

[31] Fentahun N., Belachew T., Lachat C. (2016). Determinants and morbidities of multiple anthropometric deficits in southwest rural Ethiopia. Nutrition.

[32] Gonete A.T., Alemu T.G., Mekonnen E.G., Takele W.W. (2021). Malnutrition and contributing factors among newborns delivered at the University of Gondar Hospital, Northwest Ethiopia: A cross-sectional study. BMJ Open.

[33] Kassie G.W., Workie D.L. (2019). Exploring the association of anthropometric indicators for under-five children in Ethiopia. BMC Public Health.

[34] Khamis A.G., Mwanri A.W., Kreppel K., Kwesigabo G. (2020). The burden and correlates of childhood undernutrition in Tanzania according to composite index of anthropometric failure. BMC Nutr..

[35] Kuwornu J.P., Amoyaw J., Manyanga T., Cooper E.J., Donkoh E., Nkrumah A. (2022). Measuring the overall burden of early childhood malnutrition in Ghana: A comparison of estimates from multiple data sources. Int. J. Health Policy Manag..

[36] Menalu M.M., Bayleyegn A.D., Tizazu M.A., Amare N.S. (2021). Assessment of prevalence and factors associated with malnutrition among under-five children in Debre Berhan town, Ethiopia. Int. J. Gen. Med..

[37] Pomati M., Nandy S. (2020). Assessing progress towards SDG2: Trends and patterns of multiple malnutrition in young children under 5 in West and Central Africa. Child Indic. Res..

[38] Shiferaw N., Regassa N. (2023). Levels and trends in key socioeconomic inequalities in childhood undernutrition in Ethiopia: Evidence from Ethiopia demographic and health surveys 2000–2019. Discov. Soc. Sci. Health.

[39] Odei Obeng-Amoako G.A., Myatt M., Conkle J., Muwaga B.K., Aryeetey R., Okwi A.L., Okullo I., Mupere E., Wamani H., Briend A. (2020). Concurrently wasted and stunted children 6–59 months in Karamoja, Uganda: Prevalence and case detection. Matern. Child Nutr..

[40] Olusanya B.O., Wirz S.L., Renner J.K. (2010). Prevalence, pattern and risk factors for undernutrition in early infancy using the WHO Multicentre Growth Reference: A community-based study. Paediatr. Perinat. Epidemiol..

[41] Roba A.A., Assefa N., Dessie Y., Tolera A., Teji K., Elena H., Bliznashka L., Fawzi W. (2021). Prevalence and determinants of concurrent wasting and stunting and other indicators of malnutrition among children 6–59 months old in Kersa, Ethiopia. Matern. Child Nutr..

[42] Sahiledengle B., Agho K.E., Petrucka P., Kumie A., Beressa G., Atlaw D., Tekalegn Y., Zenbaba D., Desta F., Mwanri L. (2023). Concurrent wasting and stunting among under-five children in the context of Ethiopia: A generalised mixed-effects modelling. Matern. Child Nutr..

[43] Workie D.L., Tesfaw L.M. (2021). Bivariate binary analysis on composite index of anthropometric failure of under-five children and household wealth-index. BMC Pediatr..

[44] Khaliq A., Wraith D., Miller Y., Nambiar-Mann S. (2021). Prevalence, trends, and socioeconomic determinants of coexisting forms of malnutrition amongst children under five years of age in Pakistan. Nutrients.

[45] Elmighrabi N.F., Fleming C.A., Dhami M.V., Elmabsout A.A., Agho K.E. (2023). A systematic review and meta-analysis of the prevalence of childhood undernutrition in North Africa. PLoS ONE.

[46] Gupta G., Sharma A.K., Choudhary T.S. (2017). Assessment of undernutrition among children below 5, using Composite Index of Anthropometric Failure (CIAF). Indian J. Community Health.

[47] Khanra P., Biswas S., Bose K. (2019). Nutritional Assessment by Composite Index of Anthropometric Failure among School Going Children of Purba Medinipur, West Bengal, India. Hum. Biol. Rev..

[48] Takele B.A., Gezie L.D., Alamneh T.S. (2022). Pooled prevalence of stunting and associated factors among children aged 6–59 months in Sub-Saharan Africa countries: A Bayesian multilevel approach. PLoS ONE.

[49] Permatasari T.A.E., Chadirin Y. (2022). Assessment of undernutrition using the composite index of anthropometric failure (CIAF) and its determinants: A cross-sectional study in the rural area of the Bogor District in Indonesia. BMC Nutr..

[50] Sen J., Mondal N. (2012). Socio-economic and demographic factors affecting the Composite Index of Anthropometric Failure (CIAF). Ann. Hum. Biol..

[51] Islam M.S., Biswas T. (2020). Prevalence and correlates of the composite index of anthropometric failure among children under 5 years old in Bangladesh. Matern. Child Nutr..

[52] Al-Sadeeq A.H., Bukair A.Z., Al-Saqladi A.-W.M. (2018). Assessment of undernutrition using Composite Index of Anthropo-metric Failure among children aged< 5 years in rural Yemen. East. Mediterr. Health J..

[53] Khara T. (2016). The Relationship Between Wasting and Stunting: Policy, Programming and Research Implications.

[54] Florêncio T.M.d.M.T., Ferreira H.d.S., França A.P.T.d., Cavalcante J.C., Sawaya A.L. (2001). Obesity and undernutrition in a very-low-income population in the city of Maceió, northeastern Brazil. Br. J. Nutr..

[55] Zhang Y.-Q., Li H., Wu H.-H., Zong X.-N. (2021). Stunting, wasting, overweight and their coexistence among children under 7 years in the context of the social rapidly developing: Findings from a population-based survey in nine cities of China in 2016. PLoS ONE.

[56] Rasheed W., Jeyakumar A. (2018). Magnitude and severity of anthropometric failure among children under two years using Composite Index of Anthropometric Failure (CIAF) and WHO standards. Int. J. Pediatr. Adolesc. Med..

[57] Khaliq A., Wraith D., Nambiar S., Miller Y. (2022). A review of the prevalence, trends, and determinants of coexisting forms of malnutrition in neonates, infants, and children. BMC Public Health.

[58] Ferreira H.d.S. (2020). Anthropometric assessment of children’s nutritional status: A new approach based on an adaptation of Waterlow’s classification. BMC Pediatr..

[59] Fongar A., Gödecke T., Qaim M. (2019). Various forms of double burden of malnutrition problems exist in rural Kenya. BMC Public Health.

[60] Zhang N., Becares L., Chandola T. (2016). Patterns and determinants of double-burden of malnutrition among rural children: Evidence from China. PLoS ONE.

[61] Rahman A., Rahman M.S. (2019). Rural-urban differentials of childhood malnutrition in Bangladesh. Int. J. Child Health Nutr..

[62] Anik A.I., Chowdhury M.R.K., Khan H.T., Mondal M.N.I., Perera N.K., Kader M. (2021). Urban-rural differences in the associated factors of severe under-5 child undernutrition based on the composite index of severe anthropometric failure (CISAF) in Bangladesh. BMC Public Health.

[63] Adler N.E., Ostrove J.M. (1999). Socioeconomic status and health: What we know and what we don’t. Ann. N. Y. Acad. Sci..

[64] McDonald C.M., Olofin I., Flaxman S., Fawzi W.W., Spiegelman D., Caulfield L.E., Black R.E., Ezzati M., Danaei G. (2013). The effect of multiple anthropometric deficits on child mortality: Meta-analysis of individual data in 10 prospective studies from developing countries. Am. J. Clin. Nutr..

