# Supplementary material for: Magnitudes of Various Forms of Undernutrition Among Children from the Composite Index of Anthropometric Failure in Sub-Saharan Africa: A Systematic Review and Meta-Analysis
Source: Nutrients. 2025 May 27;17(11):1818. doi: 10.3390/nu17111818 (PMC12157883; doi:10.3390/nu17111818)
Supplement: Supplementary file 1 [file nutrients-17-01818-s001.zip › Suplementary file S1.pdf]

Additional file S1. Database searches for Systematic Review and meta-analysis: Exploring magnitudes of various forms of child undernutrition from the composite index of anthropometric failure in sub-Saharan Africa.

Searching For Medline (EBSCOhost) **database (25/09/2023) (2012)**

	**Mesh heading**	**Number of studies**
#1	(MH "Prevalence") OR (MH "Magnitude Estimation") OR (MH "Effect Size") OR (MH "Trend Studies")	344,946
#2	Prevalence OR magnitude OR burden OR “overall burden” OR “pooled prevalence” OR “pooled magnitude”	1,481,838
#3	#1 OR #2	1,481,838
#4	(MH "Nutritional Status") OR (MH "Child Nutrition Disorders+") OR (MH "Undernutrition") OR (MH "Malnutrition+") OR (MH "Growth Disorders+") OR (MH "Nutrition Disorders+")	498,917
#5	(MH "Thinness")	7822
#6	(MH "Failure to Thrive")	2436
#7	(MH "Severe Acute Malnutrition+")	3097
#8	(MH "Anthropometry+") OR (MH "Nutritional Assessment") OR (MH "Growth+/EV")	563877
#9	(MH "Nutrition Assessment") OR (MH "Nutritional Physiological Phenomena+")	683824
#10	(MH "Starvation") OR (MH "Hunger") OR (MH "Poverty+")	65655

#11	malnutrition* OR malnourish* OR undernutrition* OR undernurish* OR “nutritional outcome*” OR “nutritional deficien*” OR “nutritional disorder” OR “insufficient nutrition” OR “nutritional status” OR “nutritional problem*” OR starvation* OR hunger OR stunt* OR “height for age” OR “length for age” OR wast* OR thinness OR “weight for height” OR underweight* OR “weight for age” OR “stunting index” OR “wasting index” OR “underweight index” OR “undernutrition ind*” OR (composite OR conventional) N1 (failure OR ind*) OR “anthropometric deficien*” OR (standalone OR single OR double OR triple OR multiple OR coexisting) N3 (failure OR undernutrition) OR “multiple undernutrition” OR “co-existing undernutrition” OR “concurr* undernutrition” OR “overlapped undernutrition” OR “anthropometric assessment*” OR “anthropometric evaluation”	521043
#12	#4 OR #5 OR #6 OR #7 OR #8 OR #9 OR #10 OR #11	1,829,583
#13	(MH "Infant Nutrition+") OR (MH "Child Nutritional Physiology+") OR (MH "Infant Nutrition Disorders") OR (MH "Infant Nutritional Physiology+")	4681
#14	(MH "Child+") OR (MH "Infant, Newborn+") OR (MH "Infant+") OR (MH "Child, Preschool")	2,789,364
#15	child* OR “young child” OR infan* OR neona* OR toddler* OR p?ediatric* OR bab* OR under N3 (five OR two) OR preschool OR kid* OR “0 to 60 month*” OR “0 to 59 month*” OR “6 to 24 month*” OR “6 to 59 month*” OR “0 to 36 month*” OR “6 to 36 month*” OR “0 to 24	5,069,520

	month*" OR "24 to 59 month*" OR "0 to 2 year*" OR "0 to 5 year*" OR "2 to 5 year*"	
#16	#13 OR #14 OR #15	5,069,520
#17	#3 AND #12 AND #16	2012

Limiter: *date 2006, age under-five, language English, geographic region sub-Saharan Africa*

Search for CINHAL (EBESCO) database (22/09/2023) (60)

prevalence OR magnitude OR burden OR estimate* OR "overall burden" OR "pooled prevalence" OR "pooled magnitude" AND malnutrition* OR malnourish* OR undernutrition* OR undernurish* OR "nutritional outcome*" OR "nutritional deficien*" OR "nutritional disorder" OR "insufficient nutrition" OR "nutritional status" OR "nutritional problem*" OR starvation* OR hunger OR stunt* OR "height for age" OR "length for age" OR wast* OR thinness OR "weight for height" OR underweight* OR "weight for age" OR "stunting index" OR "wasting index" OR "underweight index" OR "undernutrition ind*" OR (composite OR conventional) N1 (failure OR ind*) OR "anthropometric deficien*" OR (standalone OR single OR double OR triple OR multiple OR coexisting) N3 (failure OR undernutrition) OR "multiple undernutrition" OR "co-existing undernutrition" OR "concurr* undernutrition" OR "overlapped undernutrition" OR "anthropometric assessment*" OR "anthropometric evaluation" AND child* OR "young child" OR infan* OR neona* OR toddler* OR p?ediatric* OR bab* OR under N3 (five OR two) OR preschool OR kid* OR "0 to 60 month*" OR "0 to 59 month*" OR "6 to 24 month*" OR "6 to 59 month*" OR "0 to 36 month*" OR "6 to 36 month*" OR "0 to 24 month*" OR "24 to 59 month*" OR "0 to 2 year*" OR "0 to 5 year*" OR "2 to 5 year*"

Limiter: *date 2006, geographic region sub-Saharan Africa, age under-five, language English*

Search for Web of science database (22/09/2023) (1244)

prevalence OR magnitude OR burden OR estimate* OR “overall burden” OR “pooled prevalence” OR “pooled magnitude” (topic) AND malnutrition* OR malnourish* OR undernutrition* OR undernurish* OR “nutritional outcome*” OR “nutritional deficien*” OR “nutritional disorder” OR “insufficient nutrition” OR “nutritional status” OR “nutritional problem*” OR starvation* OR hunger OR stunt* OR “height for age” OR “length for age” OR wast* OR thinness OR “weight for height” OR underweight* OR “weight for age” OR “stunting index” OR “wasting index” OR “underweight index” OR “undernutrition ind*” OR (composite OR conventional) NEAR/1 (failure OR ind*) OR “anthropometric deficien*” OR (standalone OR single OR double OR triple OR multiple OR coexisting) NEAR/3 (failure OR undernutrition) OR “multiple undernutrition” OR “co-existing undernutrition” OR “concurr* undernutrition” OR “overlapped undernutrition” OR “anthropometric assessment*” OR “anthropometric evaluation” (topic) AND child* OR “young child” OR infan* OR neona* OR toddler* OR p$ediatric* OR bab* OR under NEAR/3 (five OR two) OR preschool OR kid* OR “0 to 60 month*” OR “0 to 59 month*” OR “6 to 24 month*” OR “6 to 59 month*” OR “0 to 36 month*” OR “6 to 36 month*” OR “0 to 24 month*” OR “24 to 59 month*” OR “24 to 59 month*” OR “0 to 2 year*” OR “0 to 5 year*” OR “2 to 5 year*”

Limiter: *date 2006, geographic region sub-Saharan Africa, age under-five, language English*

Searching for Scopus database (23/09/2023) (47)

(TITLE-ABS-KEY (prevalence OR magnitude OR burden OR estimate* OR “overall burden” OR “pooled prevalence” OR “pooled magnitude”) AND TITLE-ABS-KEY (malnutrition* OR malnourish* OR undernutrition* OR undernurish* OR “nutritional outcome*” OR “nutritional deficien*” OR “nutritional disorder” OR “insufficient nutrition” OR “nutritional status” OR “nutritional problem*” OR starvation* OR hunger OR stunt* OR “height for age” OR “length

for age” OR wast* OR thinness OR “weight for height” OR underweight* OR “weight for age” OR “stunting index” OR “wasting index” OR “underweight index” OR “undernutrition ind*” OR (composite OR conventional) W/1 (failure OR ind*) OR “anthropometric deficien*” OR (standalone OR single OR double OR triple OR multiple OR coexisting) W/3 (failure OR undernutrition) OR “multiple undernutrition” OR “co-existing undernutrition” OR “concurr* undernutrition” OR “overlapped undernutrition” OR “anthropometric assessment*” OR “anthropometric evaluation”) AND TITLE-ABS-KEY (child* OR “young child” OR infan* OR neona* OR toddler* OR p*ediatric* OR bab* OR under W/3 (five OR two) OR preschool OR kid* OR “0 to 60 month*” OR “0 to 59 month*” OR “6 to 24 month*” OR “6 to 59 month*” OR “0 to 36 month*” OR “6 to 36 month*” OR “0 to 24 month*” OR “24 to 59 month*” OR “24 to 59 month*” OR “0 to 2 year*” OR “0 to 5 year*” OR “2 to 5 year*”))

Limiter*: date 2006, geographic region sub-Saharan Africa, age under-five, language English*

Google Scholar via Publish (22/09/2023) (535)

undernutrition | children | under-five-years | anthropometric-failure (535)

Limiter: *Date 2006, exclude patent*
