# Supplementary material for: Magnitudes of Various Forms of Undernutrition Among Children from the Composite Index of Anthropometric Failure in Sub-Saharan Africa: A Systematic Review and Meta-Analysis
Source: Nutrients. 2025 May 27;17(11):1818. doi: 10.3390/nu17111818 (PMC12157883; doi:10.3390/nu17111818)
Supplement: Supplementary file 1 [file nutrients-17-01818-s001.zip › Suplementary file S3.pdf]

Table S1 contain tables for quality appraisal scores, subgroup analysis, meta-regression for study covariates, eggers test and sensitivity analysis result.

Table showing JBI quality appraisal score of the included studies.

**Quality assessment**		**JBI quality assessment tool (9 items)**									
		1	2	3	4	5	6	7	8	9	**Overall appraisal score**
Author	Year	Y/N/UN/NA	Y/N/UN/NA	Y/N/UN/NA	Y/N/UN/NA	Y/N/UN/NA	Y/N/UN/NA	Y/N/UN/NA	Y/N/UN/NA	Y/N/UN/NA	
Alarape et al	2022	Y	Y	Y	Y	Y	Y	y	y	y	9
Addo I et al	2023	Y	Y	Y	Y	Y	Y	Y	Y	Y	9
Indris A	2021	N	N	N	Y	Y	Y	Y	N	N	4
Amadu et al	2021	Y	Y	Y	Y	Y	Y	Y	Y	Y	9
Amusa et al	2023	Y	Y	Y	Y	Y	Y	Y	Y	Y	9
Asmare and Agmas	2022	Y	Y	Y	Y	Y	Y	Y	Y	Y	9
Asoba et al	2019	Y	Y	Y	Y	Y	Y	Y	Y	Y	9
Berra W	2020	UN	UN	N	Y	Y	Y	N	Y	Y	5
Gonete AT, et al	2021	N	N	N	Y	Y	Y	Y	Y	Y	6
Menalu et a	2021	UN	Y	N	Y	Y	Y	N	N	UN	4
B. O. Olusanya et al.	2010	N	N	Y	Y	Y	Y	N	Y	Y	6
M. Pomati, S. Nandy	2020	Y	Y	Y	Y	Y	Y	Y	Y	Y	9
Bidira	2021	N	Y	N	Y	Y	Y	Y	Y	Y	7
Chikhungu	2022	Y	Y	Y	Y	Y	Y	Y	Y	Y	9

Endris	2017	Y	Y	Y	Y	Y	Y	Y	Y	Y	9
Fenta	2021	Y	Y	Y	Y	Y	Y	Y	Y	Y	9
Fentahun	2016	Y	Y	N	Y	Y	Y	Y	Y	Y	8
Kassie	2019	Y	Y	Y	Y	Y	Y	Y	Y	Y	9
Khamis	2020	Y	Y	Y	Y	Y	Y	Y	Y	Y	9
Kuwornu	2022	Y	Y	Y	Y	Y	Y	Y	Y	Y	9
Ziba	2018	Y	Y	Y	Y	Y	Y	Y	Y	Y	9
Shiferaw	2023	Y	Y	Y	Y	Y	Y	Y	Y	Y	9
OdeiObeng-Amoako	2020	Y	Y	Y	Y	Y	Y	Y	Y	Y	9
Roba	2021	Y	Y	Y	Y	Y	Y	Y	Y	Y	9
Sahiledengle	2023	Y	Y	Y	Y	Y	Y	Y	Y	Y	9
Workie	2021	Y	Y	Y	Y	Y	Y	Y	Y	Y	9
M.Ziba	2018	Y	Y	Y	Y	Y	Y	Y	Y	Y	9

NB scoring for each study will be given as 1 for “yes” and 0 for response of “no, unclear and not applicable”.

Abbreviation: Y, yes; N, no; NA, not applicable; UN, unclear; IN, include EX, exclude; SFI, seek further information, JBI, Joana Briggs Institute

JBI critical appraisal checklist for prevalence report

1. Was the sample frame appropriate to address the target population?
2. Were study participants sampled in an appropriate way?
3. Was the sample size adequate?
4. Were the study subjects and the setting described in detail?
5. Was the data analysis conducted with sufficient coverage of the identified sample?
6. Were valid methods used for the identification of the condition?
7. Was the condition measured in a standard, reliable way for all participants?
8. Was there appropriate statistical analysis?
9. Was the response rate adequate, and if not, was the low response rate managed appropriately?
