# Supplementary material for: Magnitudes of Various Forms of Undernutrition Among Children from the Composite Index of Anthropometric Failure in Sub-Saharan Africa: A Systematic Review and Meta-Analysis
Source: Nutrients. 2025 May 27;17(11):1818. doi: 10.3390/nu17111818 (PMC12157883; doi:10.3390/nu17111818)
Supplement: Supplementary file 1 [file nutrients-17-01818-s001.zip › Suplementary file S4.pdf]

Table S2 showing the meta-regression analysis for the various categories of undernutrition by study covariates.

Table showing the meta-regression analysis for the various categories of undernutrition by study covariates.

**Study covariates**	**CIAF**		**Stunting only**		**Wasting only**		**Underweight only**		**SU**		**WU**		**SWU**	
	**Meta-regression coefficient (95%CI)**	**P-value**	**Meta-regression coefficient (95%CI)**	**p-value**	**Meta-regression coefficient (95%CI)**	**P-value**	**Meta-regression coefficient (95%CI)**	**P-value**	**Meta-regression coefficient (95%CI)**	**P-value**	**Meta-regression coefficient (95%CI)**	**P-value**	**Meta-regression coefficient (95%CI)**	**P-value**
**Study year**	-4·16(-6·73, -1·60)	**0·005***	-1·90(-9·00, 5·18)	0·49	-0·10(-0·92 ·71)	0·76	1·09(-1·29, 3·47)	0·31	0·36(-3·15, 3·88)	0·78	-0·47(-1·29, 0·33)	0·20	-0·24(-0·69, 0·20)	0·23
**Country**	1·30(-1·15, 3·75)	0·26	3·44(-1·42, 8·32)	0·12	-0·16(-1·10, 0·78)	0·68	0·10(-2·74, 2·94)	0·93	-0·39(-2·82, 2·02)	0·67	-0·39(-1·35, 0·56)	0·35	-0·294 (-0·79, 0·20)	0·21
**Age group**	5·33(-1·11, 11·77)	0·09	1·05(-19·02, 21·14)	0·89	0·34(-1·62, 2·32)	0·68	-3·50(-9·41, 2·40)	0·19	-1·58(-11·59, 8·42)	0·68	1·13(-0·89, 3·15)	0·22	1·81(-0·28, 3·92)	0·08
**Sub-region**	--7·59(-12·31, -2·88)	**0·005***	-108(-31·48,9·87)	0·19	-0·71(-2·26, 0·83)	0·30	0·78(-3·79, 5·37)	0·68	-1·52(-8·42, 5·37)	0·57	-0·14(-1·71, 1·41)	0·83	-0·91(-1·73, 0·10)	**0·03***
**Data sources**	11·58(-9·94, 33·11)	0·25	NA	-	NA	-	NA	-	-	-	-	-	-1·94(-6·63, 2·73)	0·35
**Sample size**	-1·55(-2·93, -0·17)	**0·031***	-2·19(-5·49, 1·09)	0·14	-0·38(-0·94, 0·18)	0·15	-0·47 (-2·17, 1·21)	0·51	-0·27(-1·91, 1·36)	0·66	-0·28(-0·86, 0·29)	0·27	-0·27 (-0·53, -0·011	**0·043***
**Quality score**	-18·25()	0·08	-17·08(-123·37, 89·20)	0·67	1·79(-4·42, 8·01)	0·51	4·33(-12·56, 21·24)	0·55	0·72(--52·06, 53·51)	0·97	-2·09(-8·03, 3·83)	0·42	0·54(-7·69, 8·79)	0·89

***pvalue≤0·05, CIAF=composite index of anthropometric failure, SU=stunting with underweight, WU=wasting with underweight, SWU=stunting &wasting with underweight.**
