# Supplementary material for: Magnitudes of Various Forms of Undernutrition Among Children from the Composite Index of Anthropometric Failure in Sub-Saharan Africa: A Systematic Review and Meta-Analysis
Source: Nutrients. 2025 May 27;17(11):1818. doi: 10.3390/nu17111818 (PMC12157883; doi:10.3390/nu17111818)
Supplement: Supplementary file 1 [file nutrients-17-01818-s001.zip › Suplementary file S5.pdf]

Figure S2-S8 contains funnel plot for visual asymmetry tests of publication biases.

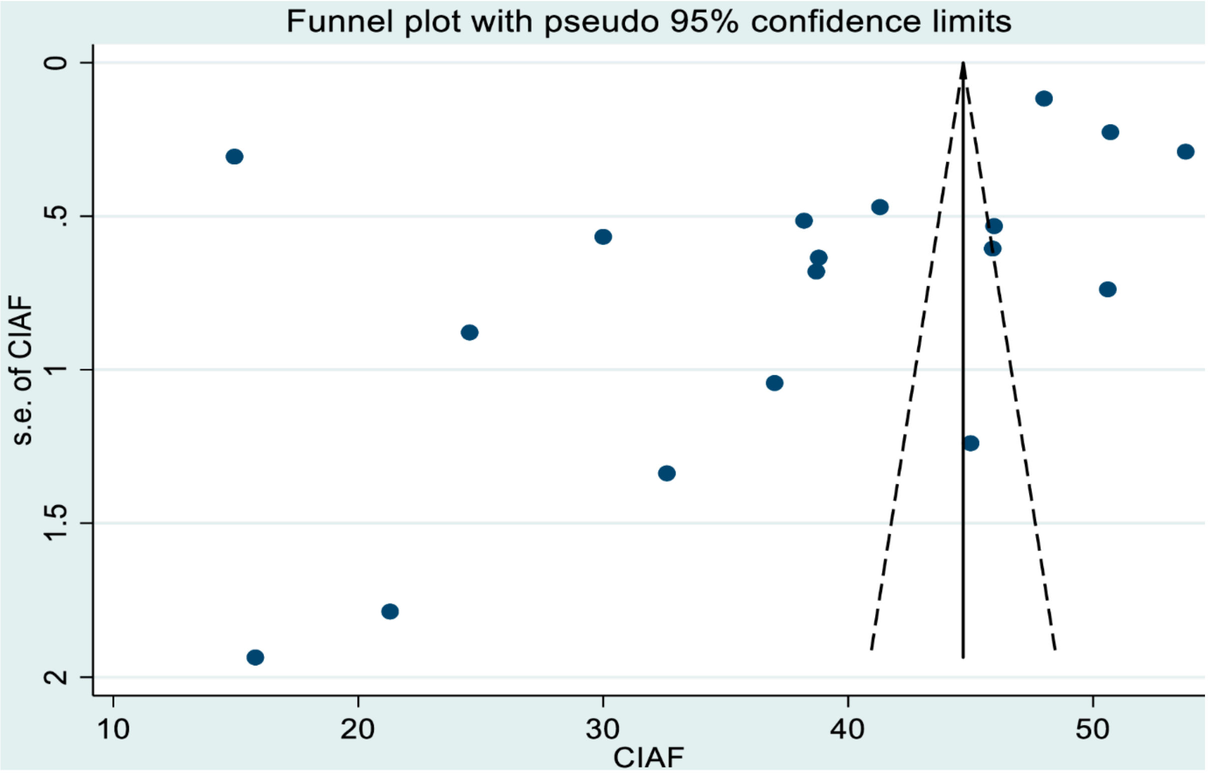

Figure S2. Funnel plot for assessing the publication bias of studies included for CIAF

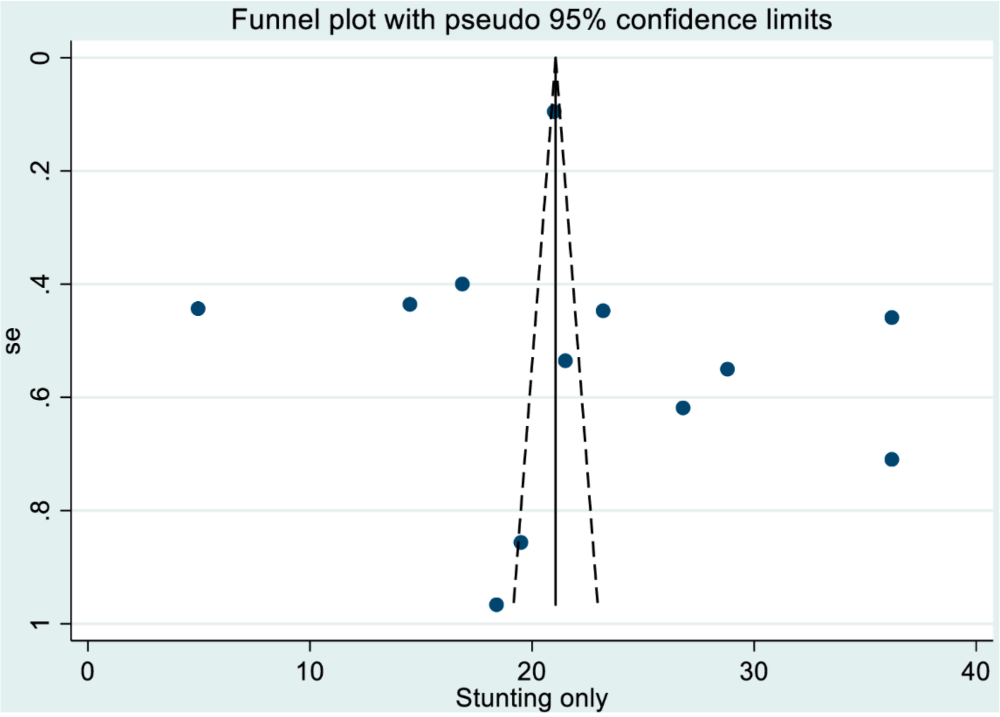

Figure S3. Funnel plot for assessing the publication bias of studies included for stunting only

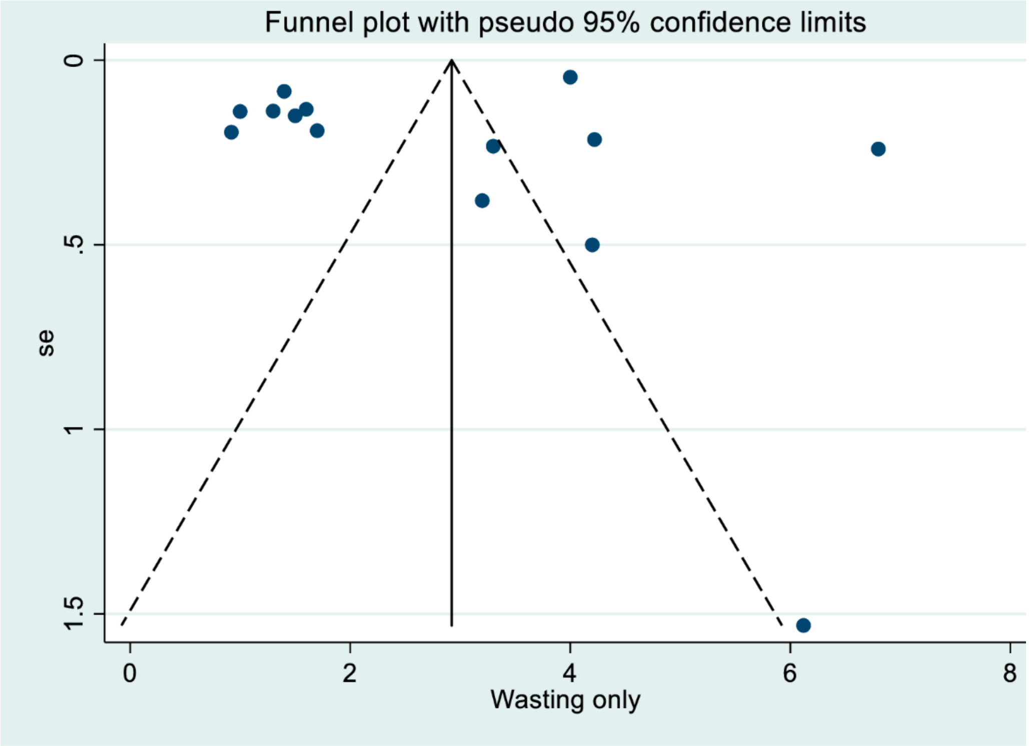

Figure S4. Funnel plot for assessing the publication bias of studies included for wasting only

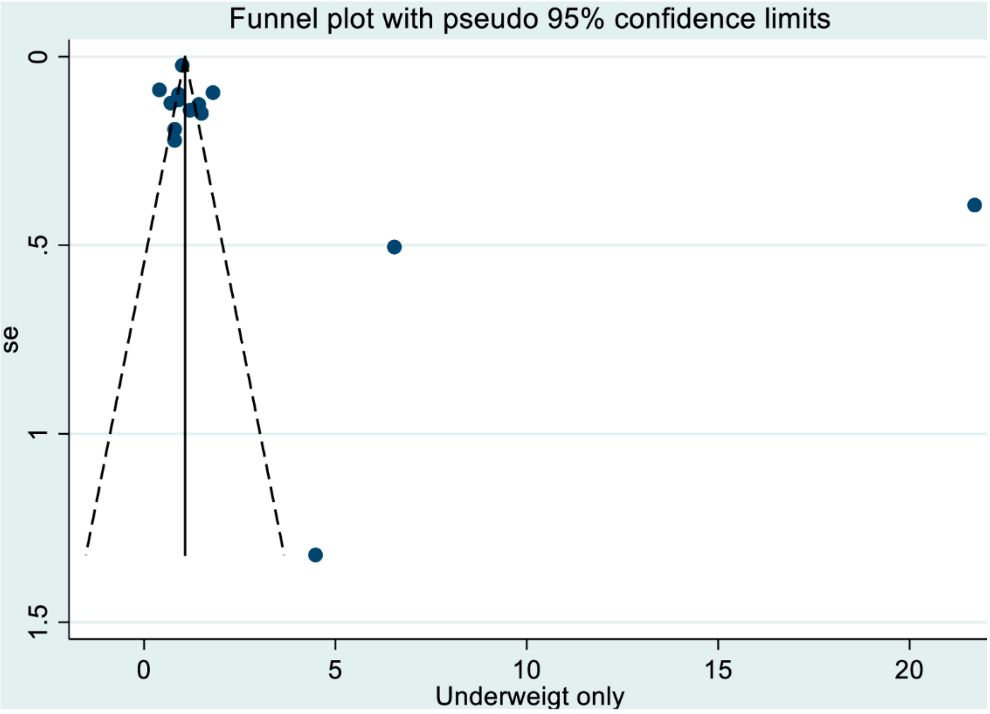

Figure S5. Funnel plot for assessing the publication bias of studies included for underweight only

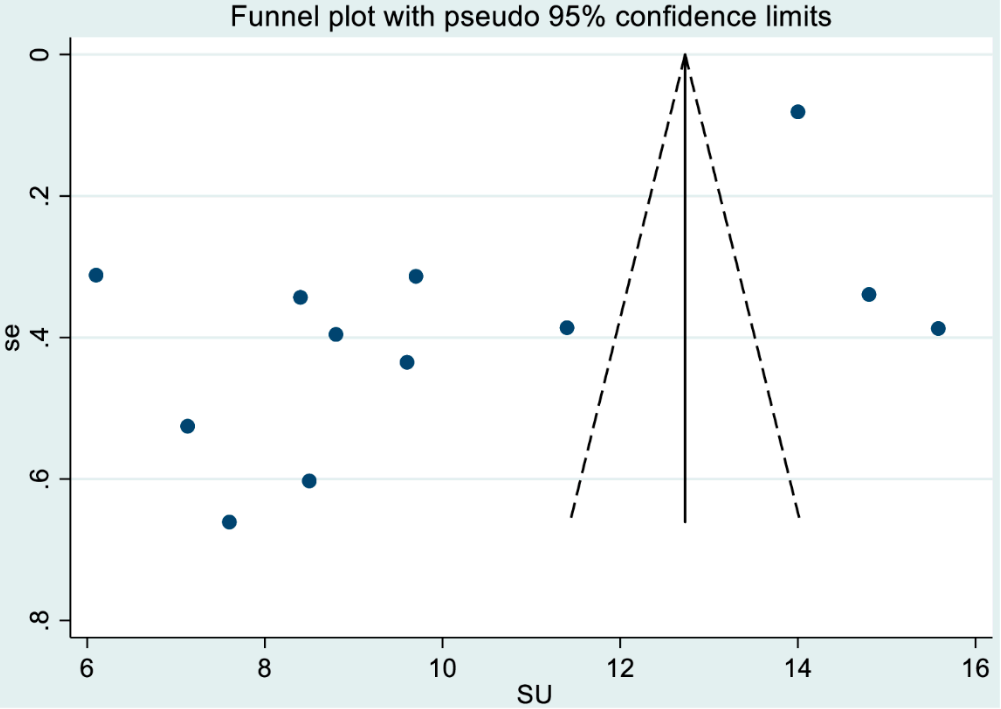

Figure S6. Funnel plot for assessing the publication bias of studies included for stunting with underweight

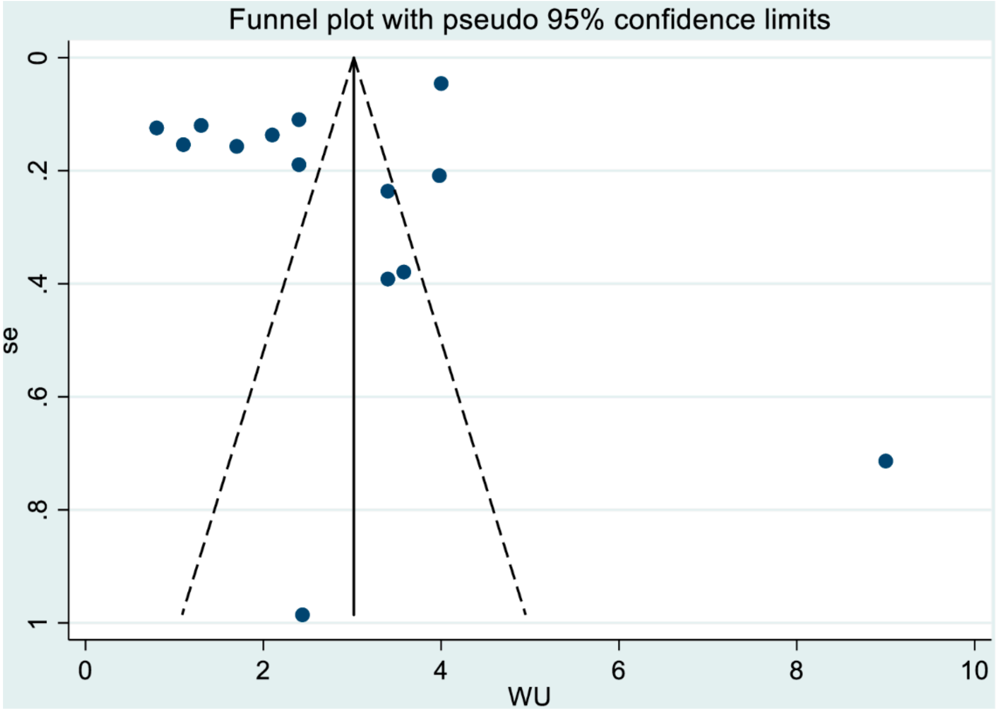

Figure S7. Funnel plot for assessing the publication bias of studies included for concurrent wasting-underweight

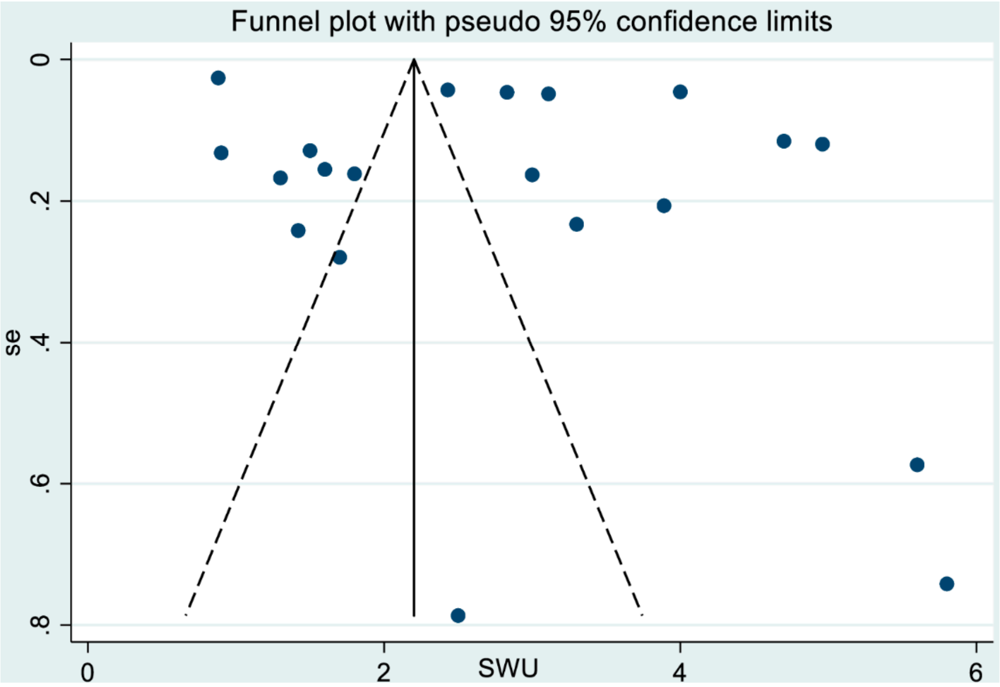

Figure S8. Funnel plot for assessing the publication bias of studies included for triple coexistence of undernutrition
