# Supplementary material for: Magnitudes of Various Forms of Undernutrition Among Children from the Composite Index of Anthropometric Failure in Sub-Saharan Africa: A Systematic Review and Meta-Analysis
Source: Nutrients. 2025 May 27;17(11):1818. doi: 10.3390/nu17111818 (PMC12157883; doi:10.3390/nu17111818)
Supplement: Supplementary file 1 [file nutrients-17-01818-s001.zip › Suplementary file S6.pdf]

Table S3 sensitivity analysis after removing studies with low quality appraisal score.

Comparison of pooled estimates of various categories of undernutrition after removing studies with low quality appraisal score							
	**CIAF**	**Stunting only**	**Wasting only**	**Underweight only**	**SU**	**WU**	**SWU**
Before	37·45% (95% CI:31·97,42·92), I^2=99·9%, P <0·00	22·32% (95% CI 18·26–26·39), I^2=99·7%, P <0·00	2·83% (95% CI 1·94–3·72), I^2=99·3%, P <0·00	3·02% (95% CI 2·17–3·88), I^2=99·6%, P <0·00	10·52% (95%CI 8·71–12·33), I^2=99·2%, P <0·00	2·90 (95% CI 2·11–3·69), I^2=99·1%, P <0·00	2·82% (95% CI 2·19–3·44), I^2=99·7%, P <0·00
After	39·82% (95%CI 33·82–45·82), I^2=99·9%, P <0·00	22·40% (95%CI 17·98–26·82), I^2=99·7%, P <0·00	2·64% (95% CI 1·67–3·60), I^2=99·4%, P <0·00	3·11% (95% CI 2·16–4·05) I^2=99·6%, P <0·00	10·52% (95%CI 8·71–12·33), I^2=99·00%, P <0·00	2·88% (95%CI 2·02–3·74), I^2=99·2%, P <0·00	2·80% (95%CI 2·14–3·46), I^2=99·7%, P <0·00

CIAF=composite index of anthropometric failure, SU=stunting with underweight, WU=wasting with underweight, SWU=stunting wasting underweight
