# Supplementary figures and images for: Magnitudes of Various Forms of Undernutrition Among Children from the Composite Index of Anthropometric Failure in Sub-Saharan Africa: A Systematic Review and Meta-Analysis
Source: Nutrients. 2025 May 27;17(11):1818. doi: 10.3390/nu17111818 (PMC12157883; doi:10.3390/nu17111818)

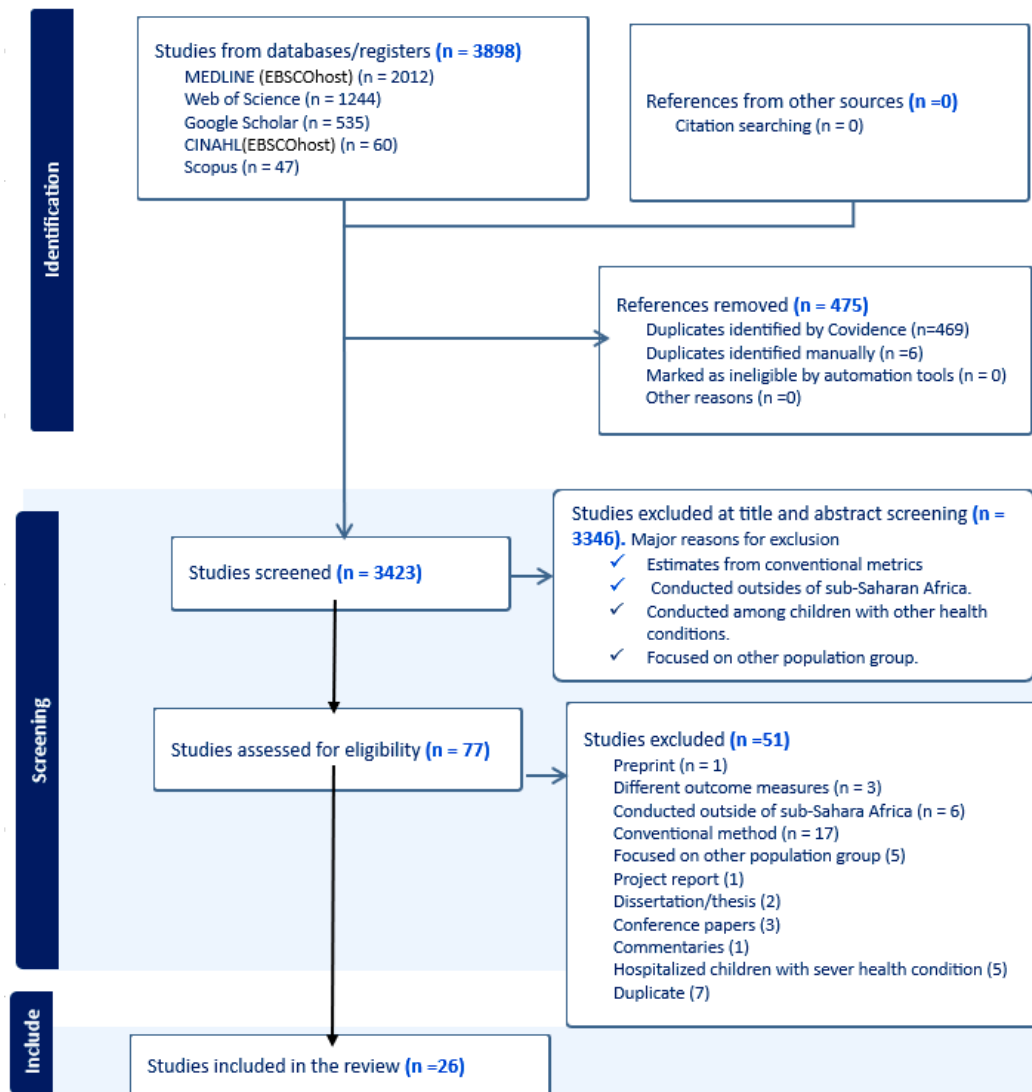

**Figure S1.** Flow diagram of the included studies.

Supplement: Supplementary file 1 [file nutrients-17-01818-s001.zip › Suplementary file S2.pdf]
